# Pareto Optimal Weight Learning and Gradient Anisotropic Supervoxel Segmentation for Thermo-Geometric Point Clouds

**DOI:** 10.3390/s26092582

**Published:** 2026-04-22

**Authors:** Tan Xutong, Chun Yin, Xuegang Huang, Xiao Peng, Junyang Liu

**Affiliations:** 1 School of Automation Engineering, University of Electronic Science and Technology of China, Chengdu 611731, China; 2 National Key Laboratory of Aerospace Physics in Fluids, Mianyang 621000, China

**Keywords:** point cloud segmentation, thermo-geometric combination, supervoxel segmentation, multi-objective optimization, multi-modal fusion

## Abstract

The simultaneous analysis of geometric morphology and thermodynamic states from heterogeneous sensing modalities is essential for high-temperature industrial inspection. While supervoxel segmentation is effective for extracting fine structures, conventional fixed-weighting schemes often struggle with the inherent heterogeneity between spatial sensors and thermal sensors. This paper proposes a segmentation framework for thermo-geometric point clouds based on Pareto-optimal weight learning and gradient anisotropy. A multi-objective evolutionary optimization algorithm is employed for multi-modal Pareto weight learning to adaptively balance geometric and thermal constraints. The developed gradient-anisotropic supervoxel generation algorithm introduces a local saliency factor to achieve fine-grained thermodynamic segmentation. Furthermore, a gradient damping mechanism is implemented to ensure high thermal-boundary adherence even in geometrically planar regions by imposing anisotropic penalty forces. Finally, a region-growing method based on the optimized multi-sensor fusion weights is utilized to merge similar supervoxels. Experimental results demonstrate that our approach outperforms traditional baselines by achieving high-fidelity thermal segmentation and multi-modal boundary preservation, while accepting a modest and necessary compromise in geometric compactness to accommodate spatial–thermal inconsistencies.

## 1. Introduction

In fields such as aerospace, energy propulsion, and advanced manufacturing, the real-time monitoring of critical structures operating under extreme high-temperature conditions imposes severe demands on advanced sensing technologies. The operational safety and reliability of these components strictly depend on high-fidelity sensor data to evaluate performance and service life [1,2]. For instance, rocket engine nozzle components operate under harsh conditions such as high-temperature gas heating, where material environments exceeding 1000 °C can induce full-field deformation [3]. Similarly, during high-speed cruise, the windward structures of aircraft experience severe aerodynamic heating, with local peak temperatures surpassing 2000 °C. Hot-end components, including aero-engines, rocket nozzles, and combustion chambers, must withstand the coupled thermo-mechanical effects of temperatures at or above 2000 °C. Under such operating conditions, the temperature magnitude and its spatial distribution not only impair the mechanical properties and thermal stability of materials but also accelerate degradation processes such as oxidation, creep, and thermal fatigue, significantly shortening the service life of critical structural members [4,5]. Consequently, industrial inspection requirements are fundamentally shifting away from traditional single-point sensing mechanisms (e.g., isolated thermocouples or localized topographical probes) toward comprehensive, full-field multi-sensor detection arrays [6]. This paradigm shift requires the seamless integration of spatial 3D sensors and thermal imaging modalities to enable the coupled analysis of thermo-geometric point clouds. Therefore, the development of integrated multi-sensor measurement systems for 3D morphology and thermal field measurement in high-temperature environments is of paramount importance for material safety monitoring, structural design optimization, and equipment operational reliability.

In these complex high-temperature scenarios, relying on a single sensing modality is insufficient for holistic quality control. For instance, geometric distortion in metal additive manufacturing components is often a physical manifestation of localized heat accumulation, whereas surface cracks in turbine blades frequently serve as precursors to abnormal thermal stress concentration [7,8]. Consequently, executing heterogeneous multi-sensor data fusion that integrates both geometric morphology sensing and thermal sensing is crucial for accurate lifetime prediction and defect detection. By mapping thermal sensor data onto a geometric model acquired by spatial sensors, thermo-geometric point clouds are generated, denoted as $P=\{p_{i}|\left(x,y,z,n,T\right)\}$. This fused representation provides a foundational cornerstone for comprehensive physical-material analysis, supporting “shape-performance integration” evaluations. Unlike 2D thermal imaging, which lacks depth information, or pure 3D models, which lack physical state data, this integrated multi-sensor representation enable the precise localization of thermal anomalies on complex curved surfaces.

Although thermo-geometric point clouds provide rich information, they consist of unstructured discrete points. To extract semantic knowledge from these fused sensor outputs, point cloud segmentation is required. In the context of thermo-geometric point cloud segmentation, high-quality results must be sufficiently fine-grained to capture micro-scale defects. Crucially, the segmentation must strictly adhere to the physical boundaries of thermal gradients to prevent defect features from being averaged into the background. Compared to conventional 3D spatial sensor data, the segmentation of fused thermo-geometric sensor data faces unique challenges. First, there is significant sensing modal heterogeneity. Geometry and temperature are governed by distinct physical laws; a geometrically smooth surface to a 3D topographical sensor may exhibit drastic temperature fluctuations when viewed by a thermal sensor, creating conflicting objectives for segmentation algorithms. Second, thermal fields possess diffuse boundary characteristics. Unlike visual features captured by standard optical sensors that often present sharp edges, the temperature gradients recorded by thermal sensors are typically smooth and gradual due to thermal conduction. This inherent sensing ambiguity makes it difficult to define crisp segmentation boundaries. Third, there are conflicting gradient scales. The magnitude of geometric variation is often incompatible with that of temperature gradient changes. Balancing these two modalities to detect boundaries that are geometrically weak but thermally prominent remains a challenging optimization problem.

In recent years, point cloud segmentation has emerged as a significant research focus within the field of computer vision [9,10,11]. Among various approaches, supervoxel-based segmentation methods offer distinct advantages for fine-grained tasks due to their excellent boundary preservation performance. As a 3D extension of superpixel segmentation in image processing, supervoxel segmentation was initially applied to video and 3D image domains, as demonstrated by the work of [12,13]. Subsequently, these techniques were adapted for 3D point cloud analysis; notably, Papon et al. [14] proposed the VCCS algorithm, which utilizes octree-based voxelization followed by uniform spatial partitioning to extract initial supervoxels. The VCCS algorithm has proven highly efficient, yielding promising results on RGB-D datasets. To improve the precision of supervoxel adherence to object boundaries, Song et al. (2014) introduced Boundary Enhanced Supervoxel Segmentation (BESS) [15], a two-stage approach designed to enhance generation quality. Further advancements include [16], which developed a heuristic algorithm leveraging local information to generate adaptive-resolution supervoxels. This method operates independently of seed point selection and more effectively preserves both object boundaries and micro-structures. In the realm of deep learning, ref. [17] explored a “clustering-prediction” online 3D semantic segmentation solution, enabling efficient learning of dense 3D representations. To address density inconsistency, ref. [18] integrated multi-resolution supervoxel algorithms with a BPSO optimization scheme, enhancing performance in urban point cloud segmentation. Efficiency was further addressed by [19], which designed interval quick-sort and region-growing methods. For handling under-segmentation, ref. [20] employed point-to-plane constraints and roughness-filtered RANSAC to resolve boundary errors between non-coplanar regions. Finally, ref. [21] proposed a boundary-aware superpixel method to achieve optimal point-to-RP matching.

However, existing supervoxel segmentation algorithms typically rely on a k-means clustering framework that minimizes a weighted distance metric combining spatial and feature information. While these methods yield significant results on indoor RGB-D sensor outputs, they exhibit severe limitations when applied to industrial thermo-geometric data. First, they depend on fixed weighting schemes that fail to adapt to the dynamic heterogeneity of thermal and geometric features, frequently resulting in over-segmentation of smooth thermal regions or under-segmentation of critical defects. Furthermore, most existing supervoxel methods assume isotropic growth and lack specific gradient-protection mechanisms to handle the diffusive characteristics of thermal fields. This deficiency often causes supervoxels to erroneously bridge the boundaries between hotspots and the substrate.

To address the aforementioned issues, this paper proposes a segmentation framework for high-temperature point clouds based on Pareto-optimal metric learning and gradient-anisotropic refinement. Unlike conventional methods that rely solely on geometric distances, this approach performs multi-objective Pareto analysis within a multimodal metric space to adaptively learn the optimal balance weights between geometric structures and thermal field attributes. This overcomes the subjectivity and inefficiency inherent in parameter tuning within traditional linear weighting metrics. To address the strong gradient boundaries common on the surfaces of high-temperature objects, an anisotropic boundary correction algorithm is designed. By leveraging local saliency and gradient damping functions, an “energy wall” is constructed during the supervoxel growth process. This forces the segmentation boundaries to adhere to actual physical mutation regions, effectively resolving the under-segmentation challenges faced by traditional methods in regions that are geometrically planar yet exhibit drastic temperature differentials. Finally, a region-growing method based on the optimized weights is applied to merge similar supervoxels, thereby completing the segmentation of the thermo-geometric point clouds.

## 2. Problem Statement

### 2.1. Representation of Thermo-Geometric Point Clouds and Supervoxel Objectives

A thermo-geometric point cloud is defined as a point set $P={\left\{p_{i}\right\}}_{i=1}^{N}$, where each point $p_{i}$ is a multidimensional vector capturing both spatial and physical attributes:

(1) $$p_{i}=\left(x_{i},y_{i},z_{i},n_{i},T_{i}\right),$$

where $\left(x_{i},y_{i},z_{i}\right)\in R^{3}$ represents the spatial coordinates, $n_{i}=\left(n_{ix},n_{iy},n_{iz}\right)$ denotes the surface normal vector representing the local geometric orientation, and $T_{i}\in R$ is the temperature value acquired from sensors.

The primary objective of the supervoxel over-segmentation stage is to partition the raw point cloud into the smallest possible, internally compact clusters with similar characteristics [14,16]. The process of supervoxel segmentation involves finding a partition $S=\{S_{1},S_{2},\ldots ,S_{k}\}$ such that:

- ${⋃}_{j=1}^{k}S_{j}=P$ and $S_{a}\cap S_{b}=\emptyset$ for a≠b;
- Each subset $S_{j}$ exhibits high homogeneity in both geometric curvature and thermal distribution;
- The boundary ∂S strictly aligns with the physical gradients of the object.

Mathematically, this is equivalent to minimizing intra-class variance while maximizing boundary adherence. As established in [16], the supervoxel identification process can be abstracted as a subset selection problem. Specifically, this involves selecting *K* representative points from the original *N* points such that the dissimilarity between each point and its corresponding representative is minimized. Consequently, the over-segmentation problem is transformed into a mapping from the raw point cloud to a set of supervoxel labels: $m:\left\{p_{1},\ldots ,p_{i},\ldots ,p_{N}\right\}\to \left\{1,\ldots ,K\right\}$, where each 3D point is assigned to a specific representative point. A supervoxel $S_{k}$ is defined as the set of all 3D points whose representative label is *k*: $S_{k}=\left\{p|r\left(p\right)=k\right\}$, where r(p) denotes the representative label of point *p*. The objective of the over-segmentation stage is therefore to determine the representative assignments that minimize the internal dissimilarity within each segmentation unit:

(2) $$r\left(p\right)=\arg\min_{k}D\left(p,r_{k}\right),$$

where *D* denotes the dissimilarity metric used to quantify the similarity between two points. To optimize this assignment process, a binary representative variable $z_{i,j}$ is introduced, and the original problem is encoded into the following energy function:

(3) $$\begin{array}{c} \min_{\left\{z_{i,j}\right\}}\sum_{i=1}^{N}\sum_{j=1}^{N}z_{i,j}D\left(p_{i},p_{j}\right)\;s.t.\;z_{i,j}\in \left\{0,1\right\},\forall i,j;\;\sum_{i=1}^{N}z_{i,j}=1,\forall j \end{array}$$

In this formulation, the binary variable $z_{i,j}$ represents the assignment relationship, where $z_{i,j}=1$ indicates that point $p_{i}$ serves as the representative point and $p_{j}$ is represented by $p_{i}$.

### 2.2. Challenges in Integrated Thermal and Geometric Analysis

While geometric curvature captured by 3D spatial sensors and thermal field characteristics recorded by thermometry sensors typically exhibit correlations during steady-state heat dissipation, spatial misalignments between these heterogeneous sensor outputs frequently occur in complex industrial scenarios. For instance, the presence of subsurface defects inducing localized hotspots, surface oxidation, the delamination of thermal barrier coatings, and dynamic processes in metal additive manufacturing can all lead to inconsistencies between local geometric and thermal features.

**Figure 1:**
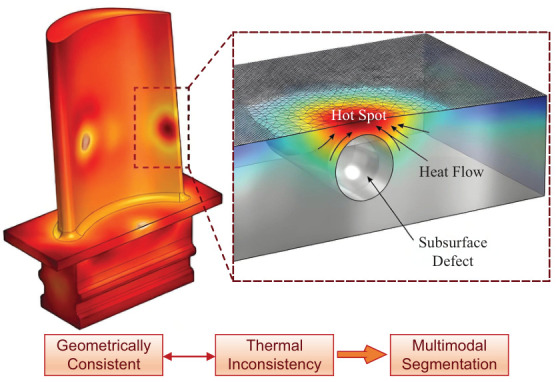
Inconsistency between geometric characteristics and thermal characteristics.

As illustrated in Figure 1, the fundamental challenge in multi-sensor data fusion lies in defining a boundary that simultaneously respects the geometric manifold and the thermal flux without exhibiting bias toward a single dominant sensing modality. Prioritizing geometric features may result in the omission of critical thermal anomalies, whereas prioritizing temperature can lead to the over-segmentation of geometric structures due to thermal sensor noise or emissivity artifacts. Conventional algorithms, such as VCCS [14] and TBPS [16], employ global fixed-weighting schemes that fail to satisfy the requirements for segmenting thermo-geometric point clouds. To address this, we introduce a multi-objective evolutionary optimization approach that simultaneously considers thermal and geometric features, dynamically optimizing weights to meet the demands of complex thermo-geometric point cloud segmentation.

## 3. Methodology

### 3.1. Multimodal Distance Metrics and Weight Learning

Traditional supervoxel segmentation algorithms rely on either a single distance metric or a weighted composite function to identify representative points. However, a single metric is insufficient for the comprehensive segmentation requirements of high-temperature point clouds, while fixed-weight composite functions lack the flexibility to adapt to complex and dynamic segmentation scenarios. To address the intricate requirements of balancing temperature, spatial distance, and geometric consistency in high-temperature turbine blades, this section introduces a multi-objective evolutionary optimization algorithm [22,23,24]. By optimizing multiple objective functions in vector form, this approach achieves Pareto-optimal results for thermo-geometric point clouds across various distance metrics.

#### 3.1.1. Definition of Multimodal Distance Metrics

(1) Spatial Dissimilarity Distance

Spatial distance is the most fundamental metric for describing the dissimilarity between two points. Drawing on the principles of VCCS [14], we restrict the search space for each representative point to its local neighborhood. This ensures that non-adjacent points are not mapped to the same supervoxel. Consequently, we establish the objective function $f_{1}$ based on spatial features:

(4) $$\begin{array}{c} f_{1}=\min_{\left\{r_{k}\right\}}\sum_{k=1}^{K}\sum_{j=1}^{\left|Ra(k)\right|}z_{k,j}D_{1}\left(r_{k},p_{j}\right),\;D_{1}\left(r_{k},p_{j}\right)={\left(x_{k}-x_{j}\right)}^{2}+{\left(y_{k}-y_{j}\right)}^{2}+{\left(z_{k}-z_{j}\right)}^{2} \end{array}$$

where *z* represents the assignment relationship, $r_{k}$ denotes the *k*-th representative point (k=1,…,K) with coordinates $r_{k}={\left[x_{k},y_{k},z_{k}\right]}^{T}$, and $p_{j}={\left[x_{j},y_{j},z_{j}\right]}^{T}$. |Ra(k)| represents the total number of points assigned to the representative point $r_{k}$.

(2) Surface Curvature Degree

Beyond spatial adjacency, the dissimilarity of normal features is a critical factor. The normal distance objective function $f_{2}$ aims to minimize the normal variation within each supervoxel, characterizing the surface curvature of a local region. If significant curvature exists within a supervoxel, it is identified as a salient feature, which contradicts the definition of a supervoxel as a collection of points with similar characteristics. Therefore, the objective function $f_{2}$ is defined as:

(5) $$\begin{array}{c} f_{2}=\min_{\left\{r_{k}\right\}}\sum_{k=1}^{K}\sum_{j=1}^{\left|Ra(k)\right|}z_{k,j}D_{2}\left(r_{k},p_{j}\right),\;D_{2}\left(r_{k},p_{j}\right)=1-\left|{\vec{N}}_{k}\;\cdot \;{\vec{N}}_{j}\right| \end{array}$$

where ${\vec{N}}_{k}=\left\{n_{x}^{k},n_{y}^{k},n_{z}^{k}\right\}$ and ${\vec{N}}_{j}=\left\{n_{x}^{j},n_{y}^{j},n_{z}^{j}\right\}$ denote the unit normal vectors of the representative point $r_{k}$ and the assigned point $p_{j}$, respectively.

(3) Thermal Dissimilarity Distance

To identify regions of thermal consistency within high-temperature point clouds, it is necessary to establish an objective function $f_{3}$ that quantifies internal temperature variations. The objective function $f_{3}$ is formulated as follows:

(6) $$\begin{array}{c} f_{3}=\min_{\left\{r_{k}\right\}}\sum_{k=1}^{K}\sum_{j=1}^{\left|Ra(k)\right|}z_{k,j}D_{3}\left(r_{k},p_{j}\right),\;D_{3}\left(r_{k},p_{j}\right)=\left|T_{k}-T_{j}\right| \end{array}$$

where $T_{k}$ and $T_{j}$ represent the temperature values of points $p_{k}$ and $p_{j}$, respectively.

#### 3.1.2. Pareto-Based Weight Optimization

Optimizing each of the dissimilarity metrics proposed in the previous section independently may fail to identify the globally optimal partition for supervoxels. Conversely, a simple linear weighting of distances often struggles to effectively balance the trade-offs between different metrics. Therefore, to determine the optimal mapping set for the point cloud, we propose optimizing the three objective functions simultaneously in vector form. The goal is to identify a set of non-dominated solutions, thereby ensuring that the resulting supervoxel segmentation scheme is Pareto-optimal.

To facilitate a multi-faceted evaluation of spatial distribution, normal variation, and thermal consistency, specifically for the segmentation of high-temperature regions in turbine blades, the multi-objective over-segmentation model is formulated as follows:

(7) $$\begin{array}{c} \min F={\left[f_{1}\left(X\right),f_{2}\left(X\right),f_{3}\left(X\right)\right]}^{T},\;s.t.\;X\in \Omega \end{array}$$

where $f_{1}$, $f_{2}$, and $f_{3}$ represent the objective functions based on spatial dissimilarity, surface curvature, and thermal difference, respectively. *X* denotes the importance weight ratio among the three metrics, and Ω represents the feasible domain of the solutions.

The MOEA/D [24,25] algorithm utilizes a series of weight vectors to decompose the original multi-objective function into a set of single-objective subproblems, optimizing these sub-targets concurrently rather than treating the multi-objective function as a monolithic entity.

Specifically, the Penalty-based Boundary Intersection (PBI) decomposition method is employed to categorize the approximation of an individual toward the optimal Pareto front into two components: the distance between the weight vector and the individual (representing distribution information) and the distance along the weight vector toward the optimal Pareto front (representing convergence information). The penalty term relaxes the constraints on the solutions and defines the ratio between diversity and convergence within the fitness function, allowing for greater flexibility when addressing complex thermo-geometric point cloud segmentation problems. The subproblem objective function based on the PBI decomposition is formulated as follows:

(8) $$\begin{array}{c} \min F\Rightarrow \min g\left(X|\vec{\beta },p*\right)=d_{1}\left(X\right)+\alpha d_{2}\left(X\right),\\ d_{1}\left(X\right)=\frac{\left|{\left(p^{*}-F\left(X\right)\right)}^{T}\;\cdot \;\vec{\beta }\right|}{\left|\vec{\beta }\right|},\;d_{2}\left(X\right)=\left|F\left(X\right)-\left(p^{*}+d_{1}\;\cdot \;\vec{\beta }\right)\right| \end{array}$$

where $\vec{\beta }={\left[\beta^{1},\beta^{2},\beta^{3}\right]}^{T}$ denotes a set of uniformly distributed weight vectors within the feasible domain, and $p^{*}$ represents the reference point vector. The parameter α serves as the penalty factor. Each individual associated with these weight vectors represents a candidate solution, allowing us to identify the Pareto-optimal supervoxel segmentation scheme within the feasible region.

**Figure 2:**
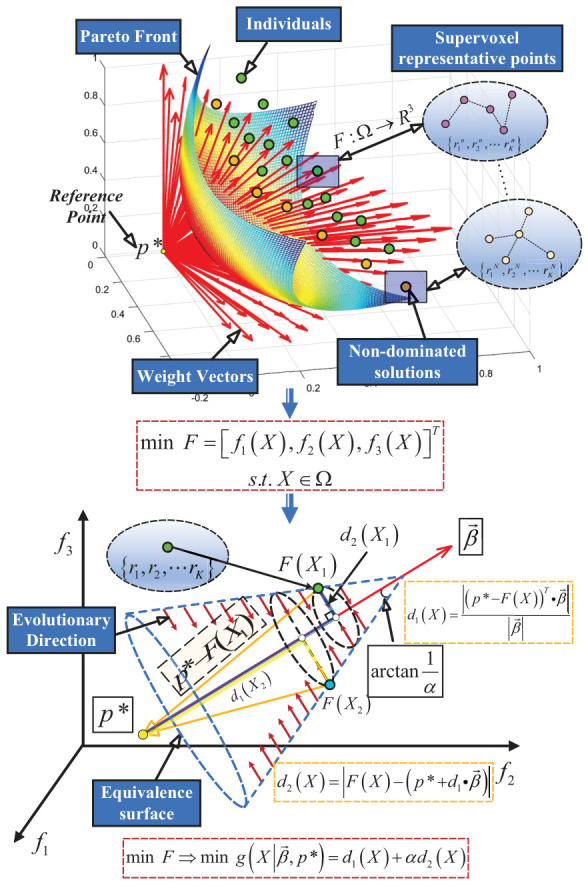
MOEA/D and population evolution.

MOEA/D employs weight vectors to guide the evolution of solutions toward the Pareto-optimal front. As illustrated in Figure 2, for the PBI subproblem $g(X|\vec{\beta },p^{*})$, $d_{1}\left(X\right)$ represents the radial distance of the individual F(X) along the weight vector $\vec{\beta }$, while $d_{2}\left(X\right)$ represents the tangential distance. The objective of the PBI decomposition is to minimize the sum of the projection distance of each individual solution along the weight vector and its perpendicular distance to that vector.

The isosurfaces of the PBI method are cone-like surfaces. These isosurfaces exert evolutionary pressure on individuals to move closer to both the weight vector $\vec{\beta }$ and the reference point $p^{*}$, thereby driving the population toward the Pareto front. For a fixed penalty factor α, consider two individuals $X_{1}$ and $X_{2}$ located on the same isosurface:

(9) $$\begin{array}{c} g\left(X_{1}|\vec{\beta },p^{*}\right)=g\left(X_{2}|\vec{\beta },p^{*}\right),\;d_{1}\left(X_{1}\right)+\alpha d_{2}\left(X_{1}\right)=d_{1}\left(X_{2}\right)+\alpha d_{2}\left(X_{2}\right) \end{array}$$

Let *L* be the distance from the reference point $p^{*}$ to the apex where the isosurface intersects the weight vector $\vec{\beta }$. Based on the principle of similar triangles:

(10) $$\frac{d_{2}\left(X_{1}\right)}{d_{2}\left(X_{2}\right)}=\frac{L-d_{1}\left(X_{1}\right)}{L-d_{1}\left(X_{2}\right)}$$

Expanding this yields:

(11) $$\begin{array}{c} L\left[d_{2}\left(X_{1}\right)-d_{2}\left(X_{2}\right)\right]=d_{2}\left(X_{1}\right)d_{1}\left(X_{2}\right)-d_{2}\left(X_{2}\right)d_{1}\left(X_{1}\right),\;L=\frac{d_{2}\left(X_{1}\right)d_{1}\left(X_{2}\right)-d_{2}\left(X_{2}\right)d_{1}\left(X_{1}\right)}{d_{2}\left(X_{1}\right)-d_{2}\left(X_{2}\right)} \end{array}$$

Defining the opening angle of the conical surface as 2θ, we have:

(12) $$\tan\theta =\frac{d_{2}\left(X_{1}\right)}{L-d_{1}\left(X_{1}\right)}$$

Substituting *L* into the equation:

(13) $$\begin{array}{c} \tan\theta =\frac{d_{2}\left(X_{1}\right)}{\frac{d_{2}\left(X_{1}\right)d_{1}\left(X_{2}\right)-d_{2}\left(X_{2}\right)d_{1}\left(X_{1}\right)}{d_{2}\left(X_{1}\right)-d_{2}\left(X_{2}\right)}-d_{1}\left(X_{1}\right)}=\frac{d_{2}\left(X_{1}\right)-d_{2}\left(X_{2}\right)}{d_{1}\left(X_{2}\right)-d_{1}\left(X_{1}\right)} \end{array}$$

Combining these yields:

(14) $$\tan\theta =\frac{1}{\alpha }$$

Based on the principle of similar triangles, the ratio of the tangential distance of F(X) to the length from the projection point on $\vec{\beta }$ to the cone apex is 1:α. Thus, the isosurface centered on the weight vector $\vec{\beta }$ is a conical surface with an opening angle of 2arctan(1/α). The PBI method drives individual evolution toward the reference point via these cone-like isosurfaces, where the penalty factor α regulates the dispersion of individuals by controlling the cone shape. A smaller α results in a larger update area, facilitating the population’s convergence toward the ideal Pareto front (PF). Conversely, a larger α restricts the update area, ensuring that updated solutions remain closer to the intersection of the weight vector and the optimal Pareto front, which enhances population diversity.

To accelerate this learning phase, the optimization is performed on a voxelized proxy of the point cloud, effectively filtering high-frequency noise and reducing computational overhead.

### 3.2. Gradient-Anisotropic Supervoxel Generation

#### 3.2.1. Supervoxel Reassignment Based on Local Saliency

Following the decomposition of the original multi-objective problem, a set of individuals $X_{l}\left(l=1,\ldots ,L\right)$ is generated, where *L* denotes the total population size. A population evolution strategy is employed to explore the Pareto solution space. Once the individuals for each generation are produced using evolutionary operators, corresponding supervoxel segmentation results must be generated for each individual based on its specific parameters.

The weight vector associated with each individual is utilized as the dynamic weighting ratio for its respective supervoxel segmentation. To achieve multi-objective supervoxel segmentation, we establish the objective function $f_{4}\left(X_{l}|{\vec{\beta }}_{X_{l}},p^{*}\right)$ as follows:

(15) $$\begin{array}{c} f_{4}\left(X_{l}|{\vec{\beta }}_{X_{l}},p^{*}\right)=\min_{\left\{r_{k}\right\}}\sum_{k=1}^{K}\sum_{j=1}^{\left|Ra(k)\right|}z_{k,j}D_{4}^{X_{l}}\left(r_{k},p_{j}\right),\\ D_{4}^{X_{l}}\left(r_{k},p_{j}\right)=\beta_{X_{l}}^{1}\;\cdot \;D_{1}\left(r_{k},p_{j}\right)+\beta_{X_{l}}^{2}\;\cdot \;D_{2}\left(r_{k},p_{j}\right)+\beta_{X_{l}}^{3}\;\cdot \;D_{3}\left(r_{k},p_{j}\right) \end{array}$$

where $X_{l}$ represents the *l*-th individual in the population, and ${\vec{\beta }}_{X_{l}}={\left[\beta_{X_{l}}^{1},\beta_{X_{l}}^{2},\beta_{X_{l}}^{3}\right]}^{T}$ denotes the weight vector corresponding to individual $X_{l}$. These weights represent the relative importance of spatial dissimilarity, surface curvature, and thermal difference, respectively, during the supervoxel extraction process. Subsequently, supervoxel extraction is performed based on this weighted multi-objective function.

**Figure 3:**
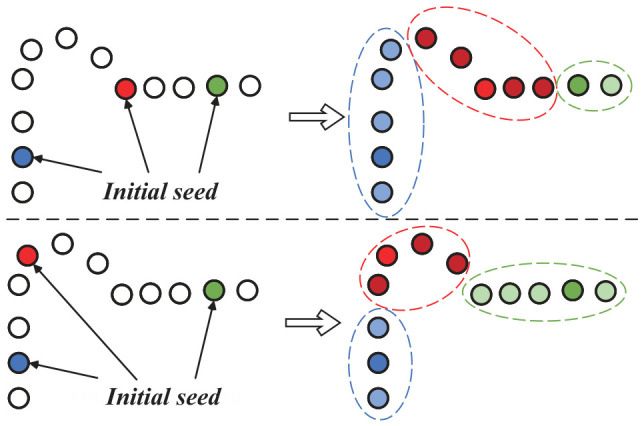
Influence of initial seed on supervoxel segmentation.

The multi-objective supervoxel segmentation is an NP-hard problem. As illustrated in Figure 3, varying seed configurations can lead to inconsistent segmentation results. For localized micro-structures, it is particularly challenging to isolate them if no initial seeds fall within their vicinity. To address this limitation, we introduce a supervoxel reassignment method based on a local saliency adaptive penalty strategy, which eliminates the dependence on initial seed points.

First, the energy function for multi-objective supervoxel segmentation is relaxed as follows:

(16) $$\begin{array}{c} f_{4}\left(X_{l}|{\vec{\beta }}_{X_{l}},p^{*}\right)=\min\sum_{i=1}^{N}\sum_{j=1}^{N}z_{i,j}D_{4}^{X_{l}}\left(p_{i},p_{j}\right)+\sum_{j=1}^{N}I\left(j\in R\right)\;\cdot \;\Omega_{j}\left(\Lambda \right),\\ D_{4}^{X_{l}}\left(p_{i},p_{j}\right)=\beta_{X_{l}}^{1}\;\cdot \;D_{1}\left(p_{i},p_{j}\right)+\beta_{X_{l}}^{2}\;\cdot \;D_{2}\left(p_{i},p_{j}\right)+\beta_{X_{l}}^{3}\;\cdot \;D_{3}\left(p_{i},p_{j}\right) \end{array}$$

where $D_{4}^{X_{l}}\left(p_{i},p_{j}\right)$ is the weighted distance function, incorporating spatial distance, surface normals, and temperature, defined by the population individual $X_{l}\;\left(l=1,\ldots ,L\right)$. The term I(j∈R) is an indicator function for the number of representative points; it takes a value of 1 if point $p_{j}$ is selected as a representative, and 0 otherwise. $\Omega_{j}\left(\Lambda \right)$ represents an adaptive penalty term based on local attribute saliency, defined as:

(17) $$\Omega_{j}\left(\Lambda \right)=\Lambda \;\cdot \;\frac{1}{1+\beta_{X_{l}}^{2}\;\cdot \;\sigma_{norm}\left(S_{j}\right)+\beta_{X_{l}}^{3}\;\cdot \;\sigma_{temp}\left(S_{j}\right)}$$

where Λ is a global regularization parameter. $\sigma_{norm}\left(S_{j}\right)$ denotes the standard deviation of surface normals within supervoxel $S_{j}$, serving as a measure of normal feature saliency, while $\sigma_{temp}\left(S_{j}\right)$ denotes the standard deviation of temperature within $S_{j}$ to quantify thermal feature saliency.

The first term in the formulation (16) ensures that the selected representative points maximally approximate the original point cloud, thereby minimizing internal variance and maintaining supervoxel compactness. The second term, the penalty term, guides the number of identified representative points toward a predefined value. Initially, a small global parameter Λ is set to encourage the algorithm to identify a larger number of representative points. During the iterative process, Λ is progressively doubled, increasing the value of the penalty term $\Omega_{j}\left(\Lambda \right)$. This ensures that the energy reduction gained from decreasing the number of representative points eventually outweighs the error increase caused by merging, thus driving the merging and reassignment process of similar supervoxels.

To account for the complex surfaces and high-temperature characteristics of turbine blades, an adaptive penalty term based on local attribute saliency is designed. This term adaptively adjusts the “merging resistance” of each supervoxel according to its specific salient features, enabling fine-grained segmentation of thermo-geometric point clouds. The rationale is that while a global regularization parameter Λ could achieve general segmentation, it often fails to isolate local fine structures, such as film cooling holes, which may be geometrically planar but exhibit distinct thermal gradients. If Λ is set too high, the algorithm tends to merge high-temperature hotspots with cooler surrounding regions to minimize total energy, leading to a loss of critical defect boundaries. Conversely, if Λ is too low, while thermal anomalies are correctly isolated, insignificant flat regions in the background become over-segmented, increasing the computational burden.

Based on these considerations, an adaptive factor measuring supervoxel saliency is appended to the global parameter Λ. In flat regions with uniform temperature, the saliency parameters for normal and temperature attributes, $\sigma_{norm}\left(S_{j}\right)$ and $\sigma_{temp}\left(S_{j}\right)$, are small, resulting in a large penalty $\Omega_{j}\left(\Lambda \right)\approx \Lambda$. This implies a high reward for merging, encouraging the generation of larger supervoxels. In contrast, at geometric edges or thermal anomaly regions, the saliency parameters are large, leading to a significantly smaller penalty $\Omega_{j}\left(\Lambda \right)\ll \Lambda$. This reduced reward for merging encourages the algorithm to retain these salient supervoxels, thereby preserving fine details and critical features.

During the multi-objective iteration process, multiple individuals require repeated evaluation of equations using distinct $\Omega_{j}\left(\Lambda \right)$ values. Given that supervoxels are local spatial entities, local information can be leveraged to accelerate the optimization. We propose an energy minimization method based on a bottom-up merging strategy.

Consider two adjacent supervoxels, $S_{i}$ and $S_{j}$, with respective representative points $r_{i}$ and $r_{j}$. If $S_{j}$ is to be merged into $S_{i}$, prior to merging, the points $p\in S_{j}$ are represented by $r_{j}$. The local energy of these two supervoxels before merging, $f_{4}^{pre}$, is defined as:

(18) $$f_{4}^{pre}=\sum_{p\in S_{i}}D_{4}^{X_{l}}\left(p,r_{i}\right)+\Omega_{i}\left(\Lambda \right)+\sum_{q\in S_{j}}D_{4}^{X_{l}}\left(q,r_{j}\right)+\Omega_{j}\left(\Lambda \right)$$

After merging, the representative $r_{j}$ is removed, and all points in $S_{j}$ are assigned to $r_{i}$. The number of representatives decreases by one, reducing the penalty cost by one unit. The new local energy $f_{4}^{after}$ is:

(19) $$f_{4}^{after}=\sum_{p\in S_{i}\cup S_{j}}D_{4}^{X_{l}}\left(p,r_{i}\right)+\Omega_{i}\left(\Lambda \right)$$

The total energy reduction, $\Delta E=f_{4}^{pre}-f_{4}^{after}$, represents the gain from merging. A positive ΔE indicates that the merge is energetically favorable:

(20) $$\begin{array}{cc} \Delta E & =\left(\sum_{p\in S_{i}}D_{4}^{X_{l}}\left(p,r_{i}\right)+\sum_{q\in S_{j}}D_{4}^{X_{l}}\left(q,r_{j}\right)+\Omega_{i}\left(\Lambda \right)+\Omega_{j}\left(\Lambda \right)\right)\\ & \;-\left(\sum_{p\in S_{i}}D_{4}^{X_{l}}\left(p,r_{i}\right)+\sum_{q\in S_{j}}D_{4}^{X_{l}}\left(q,r_{i}\right)+\Omega_{i}\left(\Lambda \right)\right)\\ & =\Omega_{j}\left(\Lambda \right)+\sum_{q\in S_{j}}D_{4}^{X_{l}}\left(q,r_{j}\right)-\sum_{q\in S_{j}}D_{4}^{X_{l}}\left(q,r_{i}\right) \end{array}$$

In the decision-making process, calculating $\sum_{q\in S_{j}}D_{4}^{X_{l}}\left(q,r_{i}\right)$ requires iterating over every point in $S_{j}$. For high-resolution turbine blade point clouds, which often contain millions of points, this is computationally prohibitive. To accelerate this, we invoke the triangle inequality property of the multimodal weighted distance $D_{4}^{X_{l}}$. For any three points a,b,c:

(21) $$\begin{array}{cc} D_{4}^{X_{l}}\left(a,c\right) & =\beta_{X_{l}}^{1}\;\cdot \;D_{1}\left(a,c\right)+\beta_{X_{l}}^{2}\;\cdot \;D_{2}\left(a,c\right)+\beta_{X_{l}}^{3}\;\cdot \;D_{3}\left(a,c\right)\\ & \leq \beta_{X_{l}}^{1}\left[D_{1}\left(a,b\right)+D_{1}\left(b,c\right)\right]+\beta_{X_{l}}^{2}\left[D_{2}\left(a,b\right)+D_{2}\left(b,c\right)\right]+\beta_{X_{l}}^{3}\left[D_{3}\left(a,b\right)+D_{3}\left(b,c\right)\right]\\ & =D_{4}^{X_{l}}\left(a,b\right)+D_{4}^{X_{l}}\left(b,c\right) \end{array}$$

Thus, for any point $q\in S_{j}$, it follows that $D_{4}^{X_{l}}\left(q,r_{i}\right)\leq D_{4}^{X_{l}}\left(q,r_{j}\right)+D_{4}^{X_{l}}\left(r_{j},r_{i}\right)$. Summing over all points in $S_{j}$:

(22) $$\sum_{q\in S_{j}}D_{4}^{X_{l}}\left(q,r_{i}\right)\leq \sum_{q\in S_{j}}D_{4}^{X_{l}}\left(q,r_{j}\right)+\left|Ra\left(j\right)\right|\;\cdot \;D_{4}^{X_{l}}\left(r_{j},r_{i}\right)$$

where |Ra(j)| is the cardinality of $S_{j}$. Substituting this inequality into the ΔE expression:

(23) $$\begin{array}{cc} \Delta E & \geq \Omega_{j}\left(\Lambda \right)+\sum_{q\in S_{j}}D_{4}^{X_{l}}\left(q,r_{j}\right)-\left(\sum_{q\in S_{j}}D_{4}^{X_{l}}\left(q,r_{j}\right)+\left|Ra\left(j\right)\right|\;\cdot \;D_{4}^{X_{l}}\left(r_{j},r_{i}\right)\right)\\ \Delta E & \geq \Omega_{j}\left(\Lambda \right)-\left|Ra\left(j\right)\right|\;\cdot \;D_{4}^{X_{l}}\left(r_{j},r_{i}\right) \end{array}$$

To ensure that each merge operation proceeds monotonically toward energy minimization, we maintain ΔE>0 by satisfying the following condition:

(24) $$\Omega_{j}\left(\Lambda \right)>\left|Ra\left(j\right)\right|\;\cdot \;D_{4}^{X_{l}}\left(r_{j},r_{i}\right)$$

This method reduces the computational complexity from O(|Ra(j)|) to O(1). Algorithm 1 details the procedural flow of the bottom-up supervoxel reassignment. For each individual $X_{l}$ in the population, supervoxel initialization is performed based on its corresponding weight vector ${\vec{\beta }}_{X_{l}}={\left[\beta_{X_{l}}^{1},\beta_{X_{l}}^{2},\beta_{X_{l}}^{3}\right]}^{T}$. The saliency parameters $\sigma_{norm}\left(p_{i}\right)$ and $\sigma_{temp}\left(p_{i}\right)$ are pre-computed once outside the iterative loop.

The initial global penalty $\Lambda_{0}$ is determined using the median of the multi-objective weighted distances between each point and its neighbors:

(25) $$\Lambda_{0}=\mathrm{MED}\left\{\min\left(\sum_{o=1}^{3}\beta_{X_{l}}^{o}\;\cdot \;D_{o}\left(p,N_{p}\right)\right)\right\}$$

where $N_{p}$ denotes the set of neighborhood points for point *p*. During the iteration, the algorithm searches for adjacent supervoxels for each current segment, computes the adaptive penalty $\Omega_{j}\left(\Lambda \right)$ based on attribute saliency, and evaluates the merging condition:

(26) $$\Omega_{j}\left(\Lambda \right)>\left|Ra\left(j\right)\right|\;\cdot \;D_{4}^{X_{l}}\left(r_{j},r_{i}\right)$$

Following each round of merging, the global penalty is updated via Λ=Λ×γ (typically with γ=2) to exert evolutionary pressure on the total count of supervoxels to converge toward the target number *K*. The core optimization philosophy is to prioritize the aggregation of points situated within smooth regions across all feature dimensions, which supervises the supervoxels to avoid boundary crossing. By utilizing the adaptive penalty $\Omega_{j}\left(\Lambda \right)$ to quantify the feature saliency of each supervoxel, the algorithm imposes greater resistance against merging high-temperature anomaly regions with significant thermal gradients or blade edges with sharp normal variations. Consequently, both geometric details and physical thermodynamic quantities are preserved. The algorithm terminates automatically when the total number of supervoxels reduces to *K*, eliminating the need for predefined iteration limits or upper bounds on Λ.
**Algorithm 1** Supervoxel Reassignment Algorithm**Input:** Point cloud P, target number of supervoxels *K*, weight vector ${\vec{\beta }}_{X_{l}}={\left[\beta_{X_{l}}^{1},\beta_{X_{l}}^{2},\beta_{X_{l}}^{3}\right]}^{T}$
**Output:** Representative point set *R* and label set *Z*
  1:Initialization: R←P (each point is initialized as a supervoxel); Construct adjacency graph; Calculate local saliency parameters $\sigma_{norm}\left(p_{i}\right)$ and $\sigma_{temp}\left(p_{i}\right)$ for all $p_{i}\in P$; Initialize global penalty multiplier $\Lambda \leftarrow \Lambda_{0}$;  2:**while** |R|>K **do**  3:   **for** each supervoxel $S_{j}\in R$ **do**  4:     Identify the nearest neighbor supervoxel $S_{i}$;  5:     Calculate adaptive penalty $\Omega_{j}\left(\Lambda \right)$ using:$$\Omega_{j}\left(\Lambda \right)=\Lambda \;\cdot \;\frac{1}{1+\beta_{X_{l}}^{2}\sigma_{norm}\left(S_{j}\right)+\beta_{X_{l}}^{3}\sigma_{temp}\left(S_{j}\right)}$$  6:     **if** $\Omega_{j}\left(\Lambda \right)>\left|Ra\left(j\right)\right|\;\cdot \;D_{4}^{X_{l}}\left(r_{j},r_{i}\right)$ **then**  7:        $S_{i}\leftarrow S_{i}\cup S_{j}$;  8:        Ra(i)←Ra(i)+Ra(j);  9:        Update labels *Z* for all $p\in S_{j}$;10:        $R\leftarrow R\setminus \{r_{j}\}$;11:     **end if**12:   **end for**13:   Λ←Λ×γ;14:**end while**15:**return** R,Z


#### 3.2.2. Anisotropic Supervoxel Boundary Correction Based on Gradient Damping

Although the supervoxel reassignment operation effectively reduces the solution space and establishes the topological structure of the supervoxels, its bottom-up greedy strategy inevitably introduces local inconsistencies and staircase artifacts at the boundaries. These issues compromise the integrity of the supervoxels and the continuity of the boundaries. To address this, this section proposes an anisotropic supervoxel boundary correction method based on gradient damping. This method acts as a local discrete gradient descent, reassigning unstable boundary points to their nearest representative points through iteration.

Considering the feature preservation requirements of multimodal supervoxel segmentation, an anisotropic damping coefficient is introduced to ensure that supervoxel fusion does not over-smooth actual physical edges while simultaneously suppressing noise. While maintaining linear computational complexity, this approach refines the boundary properties of the supervoxels, enhancing their regularity and compactness. Furthermore, it ensures that the segments closely adhere to the actual object boundaries and thermal distribution characteristics.

Upon the conclusion of the supervoxel reassignment phase, the number of supervoxels is fixed at *K*. Consequently, the second term in the relaxed objective function $f_{4}\left(X_{l}|{\vec{\beta }}_{X_{l}},p^{*}\right)$ becomes a constant. Thus, the optimization objective simplifies to minimizing the total weighted distortion. To avoid computationally expensive global searches, we identify points situated on the interface of supervoxel boundaries. By iteratively traversing and modifying the labels of these boundary points, we seek a local solution that yields a lower energy value.

Specifically, for each point *p*, we examine its k-nearest neighbor set N(p). Point *p* is defined as a boundary point if and only if:

(27) ∃q∈N(p),s(q)≠s(p)

where s(p) denotes the supervoxel label of point *p*. In other words, if any point in the neighborhood of *p* possesses a different supervoxel label, *p* is considered a boundary point. Before the algorithm commences, a single pass is made over all points to identify those satisfying this condition, which are then pushed into a queue *Q*. Subsequent searches are restricted to the points within *Q*, significantly reducing computational complexity.

Subsequently, for each point *p* in queue *Q*, we evaluate whether reassigning *p* from its current supervoxel $S_{curr}$ to a candidate neighboring supervoxel $S_{cand}$ will result in a decrease in total energy. The energy variation ΔE is calculated as:

(28) $$\Delta E=D_{4}^{X_{l}}\left(p,r_{cand}\right)-D_{4}^{X_{l}}\left(p,r_{curr}\right)$$

where $r_{cand}$ and $r_{curr}$ are the representative points of the candidate and current supervoxels, respectively. If ΔE<0, it implies that $D_{4}^{X_{l}}\left(p,r_{cand}\right)<D_{4}^{X_{l}}\left(p,r_{curr}\right)$, indicating that point *p* is closer to the representative of the neighboring supervoxel in the multimodal weighted distance space. In such cases, the label of point *p* is updated to that of $r_{cand}$.

However, the multimodal weighted distance $D_{4}^{X_{l}}$ measures non-local differences between a point and its representative. Relying solely on $D_{4}^{X_{l}}$ may cause point *p* to frequently switch representative labels even when only minor multimodal weighted distance differences exist relative to neighboring representatives. This leads to noise sensitivity and boundary oscillation (staircase effects). To protect physical boundaries, we introduce a gradient-dependent regularization term into the local energy variation. Beyond the distance between a point and its distant representative, we incorporate the differential properties of the local surface and define the multimodal gradient field intensity G(p) at point *p*:

(29) $$G\left(p\right)=\beta_{X_{l}}^{2}\;\cdot \;∥\nabla n\left(p\right)∥+\beta_{X_{l}}^{3}\;\cdot \;∥\nabla T\left(p\right)∥$$

where ∥∇n∥ represents the normal curvature and ∥∇T∥ represents the magnitude of the temperature gradient. To achieve adaptive control, we model the potential barrier penalty as a non-linear function of the gradient intensity. We define the anisotropic damping coefficient η(p):

(30) $$\eta \left(p\right)=1+\lambda_{damp}\;\cdot \;\left(1-\exp\left(-\frac{G{\left(p\right)}^{2}}{\sigma_{g}^{2}}\right)\right)$$

where $\lambda_{damp}$ is the maximum damping gain, representing the maximum protection strength for physical boundaries (larger $\lambda_{damp}$ values preserve more boundaries), and $\sigma_{g}$ is the gradient sensitivity parameter that determines the activation threshold of the damping function. Based on this coefficient, we modify the decision inequality for boundary point exchange from $D_{4}^{X_{l}}\left(p,r_{cand}\right)<D_{4}^{X_{l}}\left(p,r_{curr}\right)$ to:

(31) $$D_{4}^{X_{l}}\left(p,r_{cand}\right)<\frac{1}{\eta (p)}\;\cdot \;D_{4}^{X_{l}}\left(p,r_{curr}\right)$$

In this formulation, η(p) acts as a penalty multiplier for the new distance $D_{4}^{X_{l}}\left(p,r_{cand}\right)$. In local regions that are flat and exhibit constant temperature, the gradient G(p)→0, causing the damping to vanish (η(p)→1). In such cases, the algorithm degrades to a simple distance-weighted refinement, allowing free label exchange to optimize geometric shape. Conversely, at sharp edges or high-temperature source boundaries where $G\left(p\right)\gg \sigma_{g}$, the damping saturates ($\eta \left(p\right)\to 1+\lambda_{damp}$). This makes the exchange extremely difficult; unless the representative $r_{cand}$ is exceptionally close to *p* across all multimodal scales, the algorithm rejects the transfer of *p* to $r_{cand}$, thereby preventing boundary sliding.

Algorithm 2 illustrates the specific steps of the supervoxel boundary correction. For each point p∈P, the multimodal gradient field G(p) is calculated once outside the loop and subsequently mapped to the damping coefficient η(p) to protect regions characterized by intense geometric and thermal variations. The multimodal weighted distances $D_{4}^{X_{l}}\left(p,r_{curr}\right)$ for all points are stored in the array Dis, enabling rapid retrieval by index during the iteration and avoiding redundant calculations.
**Algorithm 2** Anisotropic Supervoxel Boundary Correction Algorithm**Input:** Point cloud P, weight vector ${\vec{\beta }}_{X_{l}}={\left[\beta_{X_{l}}^{1},\beta_{X_{l}}^{2},\beta_{X_{l}}^{3}\right]}^{T}$, representative point set *R*, label set *Z*
**Output:** Updated label set $Z^{\prime }$
  1:Initialization:  2:For each point p∈P:  3:   Calculate the multimodal gradient field G(p);  4:   Map G(p) to the damping coefficient η(p) using the damping function;  5:   Compute the multimodal weighted distance $D_{4}^{X_{l}}\left(p,r_{curr}\right)$ to its current representative and store in array Dis;  6:   Establish neighborhood lookup table Ne and identify points with differing neighbor labels to initialize queue *Q*;  7:**while** Q≠∅ **do**  8:   Pop point $p_{j}$ from *Q*;  9:   **for** each neighbor $p_{k}\in Ne\left(j\right)$ **do**10:     **if** $r_{k}\neq r_{j}$ **then**11:        Calculate $D_{4}^{X_{l}}\left(p_{j},r_{k}\right)$;12:        **if** $D_{4}^{X_{l}}\left(p_{j},r_{k}\right)<\frac{1}{\eta (p_{j})}\;\cdot \;D_{4}^{X_{l}}\left(p_{j},r_{j}\right)$ **then**13:          Update the representative label of $p_{j}$ to $r_{k}$ and update Dis;14:          **if** $p_{k}\notin Q$ **then**15:             Append $p_{k}$ to the end of queue *Q*;16:          **end if**17:        **end if**18:     **end if**19:   **end for**20:**end while**21:**return** Updated label set $Z^{\prime }$


The algorithm identifies points whose neighborhoods contain neighbors belonging to different representative points. If such points exist, they are pushed into the queue *Q* for potential exchange. For each point $p_{j}\in Q$, the algorithm identifies neighboring points with different representative labels. It then calculates the multimodal weighted distance $D_{4}^{X_{l}}\left(p_{j},r_{k}\right)$ assuming $p_{j}$ were reassigned to representative $r_{k}$. If the magnitude of the distance reduction is sufficient to overcome the damping coefficient, the representative label of $p_{j}$ is updated to $r_{k}$. Simultaneously, the affected neighborhood points are re-added to the queue *Q*. This process iteratively refines and reassigns boundary points to their correct supervoxel sets.

### 3.3. Supervoxel Merging Based on Optimal Weights

Following the over-segmentation phase, the raw point cloud is partitioned into a set of supervoxels possessing minimal continuous features. To isolate more complete feature regions for analyzing the target high-temperature behavioral characteristics, a region-growing-based merging method is employed to identify semantically coherent segmentation results.

Given that the spatial and geometric features of voxels within a supervoxel are highly compact, the sum of local normal and curvature information can be quantified using the plane-fitting residual. Using the least squares method, a plane is fitted within each supervoxel, and the residual value is utilized to quantify the internal flatness. The supervoxel with the minimum residual value is selected as the initial seed point for the region-growing process.

Once the seed point is selected, the similarity between two supervoxels is defined to facilitate the region-growing merge. Based on the optimal non-dominated compromise solution $X_{s}$ obtained from the multi-objective supervoxel segmentation, we derive the optimal weight ratio for the current multimodal high-temperature point cloud across three metrics: spatial distance, normal difference, and thermal variance. This optimal weight ratio is then used to measure the similarity between different supervoxels:

(32) $$M\left(R_{i},R_{j}\right)=\beta_{X_{s}}^{1}\;\cdot \;|{\bar{c}}_{i}-{\bar{c}}_{j}|+\beta_{X_{s}}^{2}\;\cdot \;(1-|n_{i}\;\cdot \;n_{j}\left|\right)+\beta_{X_{s}}^{3}\;\cdot \;\left|{\bar{T}}_{i}-{\bar{T}}_{j}\right|$$

where $R_{i}$ and $R_{j}$ represent the two supervoxels under evaluation; ${\vec{\beta }}_{X_{s}}={\left[\beta_{X_{s}}^{1},\beta_{X_{s}}^{2},\beta_{X_{s}}^{3}\right]}^{T}$ is the weight vector of the Pareto-optimal compromise solution; ${\bar{c}}_{i}$ and ${\bar{c}}_{j}$ are the centroids of $R_{i}$ and $R_{j}$, respectively; $n_{i}$ and $n_{j}$ are the normal vectors derived from the plane fitting; and ${\bar{T}}_{i}=\sum_{k=1}^{|R_{i}|}T_{k}/\left|R_{i}\right|$ and ${\bar{T}}_{j}$ are the mean internal temperatures of $R_{i}$ and $R_{j}$, respectively. A smaller $M(R_{i},R_{j})$ value indicates higher similarity between the two supervoxels.

Once the initial seed points and the similarity measures between adjacent supervoxels are defined, a region-growing strategy is employed to merge supervoxels into final segmentation results. The specific steps are as follows:

- **Step 1:** Initialize the set of segmentation results S=∅, the candidate seed queue Q=∅, and the set of supervoxels *P*.
- **Step 2:** Fit a plane to the supervoxel set using the least squares method. Identify the supervoxel $P_{m}$ with the minimum fitting residual and designate it as the current active region $S_{1}=\left\{P_{m}\right\}$. Update sets such that $S=S\cup S_{1}$, $Q=Q\cup P_{m}$, and $P=P\setminus P_{m}$.
- **Step 3:** Extract a seed $q_{i}$ from the candidate queue *Q*, initialize the current region $S_{i}=\left\{q_{i}\right\}$, and retrieve its neighboring supervoxels $P_{N}=N\left(q_{i}\right)$.
- **Step 4:** Select a neighbor $P_{N}^{j}$ from $P_{N}$ and calculate the similarity $M(q_{i},P_{N}^{j})$.
- **Step 5:** If $M\left(q_{i},P_{N}^{j}\right)<Thr_{md}$, merge the current supervoxel with the seed: $S_{i}=S_{i}\cup P_{N}^{j}$ and $P=P\setminus P_{N}^{j}$.
- **Step 6:** If the fitting residual $\varepsilon (P_{N}^{j})$ of the merged supervoxel is less than the threshold φ, add it to the candidate seed queue: $Q=Q\cup P_{N}^{j}$.
- **Step 7:** Repeat Steps 4–6 until all neighboring supervoxels of the current seed $q_{i}$ have been traversed.
- **Step 8:** Append the current region to the final segmentation results: $S=S\cup S_{i}$.
- **Step 9:** Repeat Steps 3–8 until the candidate seed queue *Q* is empty.

Through this region-growing merging algorithm, the over-segmented supervoxel collection is consolidated based on feature similarity, forming large-scale segmentation regions stored in *S* that align with geometric semantics. The multi-objective supervoxel segmentation, which incorporating spatial dissimilarity, surface curvature, and thermal variance, yields results that closely adhere to the complex surface morphology of the turbine blade while capturing temperature distribution response characteristics. By utilizing the weight vector from the Pareto-optimal compromise solution, the region-growing merging process maintains high segmentation consistency with the multi-objective search results.

## 4. Results

The core focus of this study lies in the downstream algorithmic fusion of multi-modal point clouds. The upstream data acquisition was implemented as a decoupled, calibrated multi-sensor module. Specifically, the multi-camera system was calibrated using standard planar targets, view-angle discrepancies were resolved via the calibrated extrinsic matrices, and a standard Z-buffering mechanism was employed to handle potential occlusions.

Throughout the operational lifecycle of a spacecraft, turbine blades remain the most vulnerable components within the engine. These blades are not only subjected to erosion by high-temperature gases and thermal shocks from rapid temperature fluctuations but must also withstand the impact of centrifugal forces generated during high-speed rotation. The thermal stability of turbine blades is directly correlated with the overall performance of the engine.

**Figure 4:**
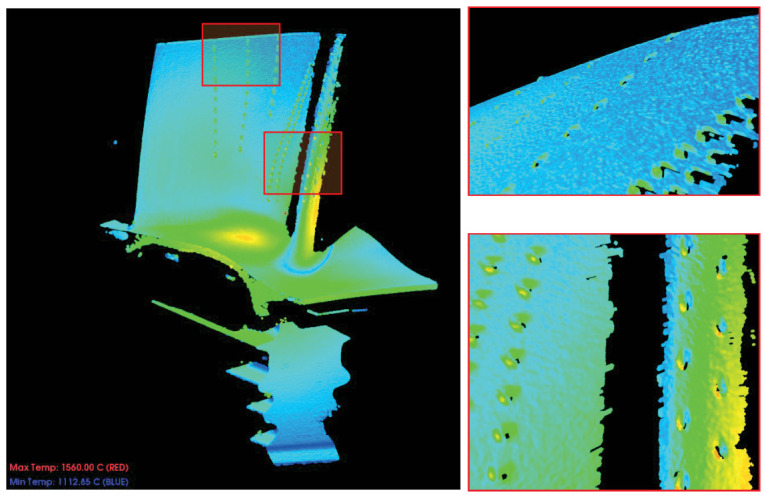
Point cloud of high temperature turbine blade.

As illustrated in Figure 4, the experimental data represents a spacecraft turbine blade during high-temperature testing. By utilizing a binocular structured light 3D sensor to capture 3D morphological data and applying a temperature mapping method, a thermo-geometric sensor data consisting of 1,057,834 points was obtained, each associated with a temperature value. It can be observed from the visualization that temperature peaks are concentrated near the blade platform, while the protruding geometries of the individual film cooling holes also form localized hotspots.

Processing million-scale high-precision turbine blade point clouds poses severe computational challenges. Specifically, initializing each point as an individual supervoxel seed for optimization is computationally intractable on the raw dense data. To ensure the efficiency of our framework, we implemented a two-tier acceleration strategy:

(1) Macro-level (Down-sampling Optimization): Based on the low-frequency characteristics of thermal fields and the scale invariance of optimal weights, a down-sampling strategy is utilized. The originally time-consuming, iteration-based multi-objective optimal weight learning process is executed on a drastically decimated down-sampled surrogate model, rendering the evolutionary search highly efficient. Ultimately, the optimal weights are transferred to the full-resolution point cloud layer to complete the point cloud segmentation.
(2) Micro-level (O(1) Bottom-up Fusion): To eliminate the bottleneck of point-wise evaluation of region merging energy in raw dense point cloud data, we leverage the mathematical properties of the distance metric. As described in Section 3.2.1, through the principle of the triangle inequality, the computational complexity of region merging validation is reduced from $O(|S_{j}|)$ to O(1). This enables million-scale point clouds to complete supervoxel segmentation within 3 s.

This dual-acceleration architecture ensures that both the parameter search phase and the final full-resolution multi-modal segmentation strictly avoid the prohibitive computational burden induced by dense point clouds.

To enhance computational efficiency, a two-layer multi-objective supervoxel segmentation approach is adopted. First, the raw high-temperature point cloud is downsampled to accelerate the multi-objective iteration speed. The total number of points after downsampling is 27,317, representing a downsampling rate of 97.42%. Multi-objective supervoxel segmentation is then executed on this downsampled cloud with the following parameters: a search resolution of 5.0, a population size P=45, a neighborhood size $Ne_{size}=7$, and a maximum of 100 iterations ($Max_{gen}=100$). The iteration counter is initialized at t=0, and the weight vector set is initialized as $B=\{{\vec{\beta }}^{1},\ldots ,{\vec{\beta }}^{45}\}$. A penalty-based decomposition method is employed to optimize the multi-objective functions. The over-segmentation algorithm is subsequently performed to identify localized regions of thermal anomalies.

To maximize the preservation of the geometric features and thermal distribution characteristics of the high-temperature blade, the multimodal gradient field intensity G(p) is first calculated to serve as the foundation for the anisotropic gradient damping. For the first individual in the population, $pop^{0}\left[1\right]$, the corresponding weight coefficients are identified, and the multimodal weighted gradient field is calculated as G(p)=0.257·∥∇n(p)∥+0.433·∥∇T(p)∥.

**Figure 5:**
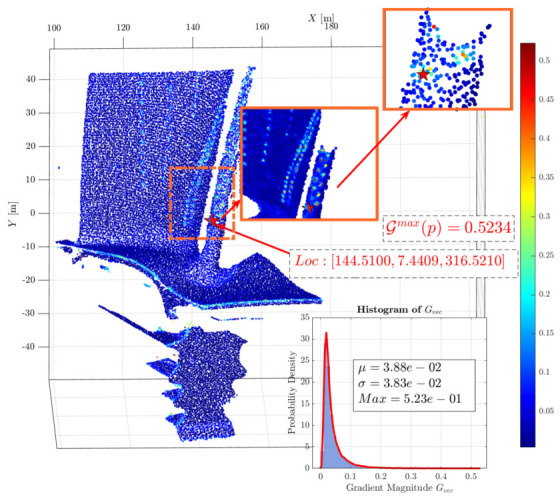
The multimodal gradient field G(p) corresponding to the individual $pop^{0}\left[1\right]$.

Figure 5 displays the multimodal weighted gradient field results for $pop^{0}\left[1\right]$. It is evident that high multimodal weighted gradients are present near the film cooling holes on the blade body and the high-curvature leading edge, representing significant variations in both normals and temperature. The maximum calculated gradient is $G^{max}\left(p\right)=0.5234$, located at coordinates [144.5100,7.4409,316.5210]. The inset in the lower-right corner provides a histogram of the gradient distribution. The overall gradient ranges from $\left[4.1496\times 10^{-3},5.2343\times 10^{-1}\right]$, with a mean of $\mu =3.88\times 10^{-2}$ and a standard deviation of $\sigma =3.83\times 10^{-2}$. This distribution indicates that most regions are relatively smooth, necessitating targeted protection and preservation of features in localized anomaly areas.

Based on the gradient field, the anisotropic damping coefficient η(p) is established to adaptively regulate the boundary exchange resistance of supervoxels across different regions, thereby preventing local anomaly areas from being over-smoothed. The maximum damping gain is set to $\lambda_{damp}=1.5$, and the gradient sensitivity parameter is defined as $\sigma_{g}^{2}={\left(\mu +2\sigma \right)}^{2}+\varepsilon$.

**Figure 6:**
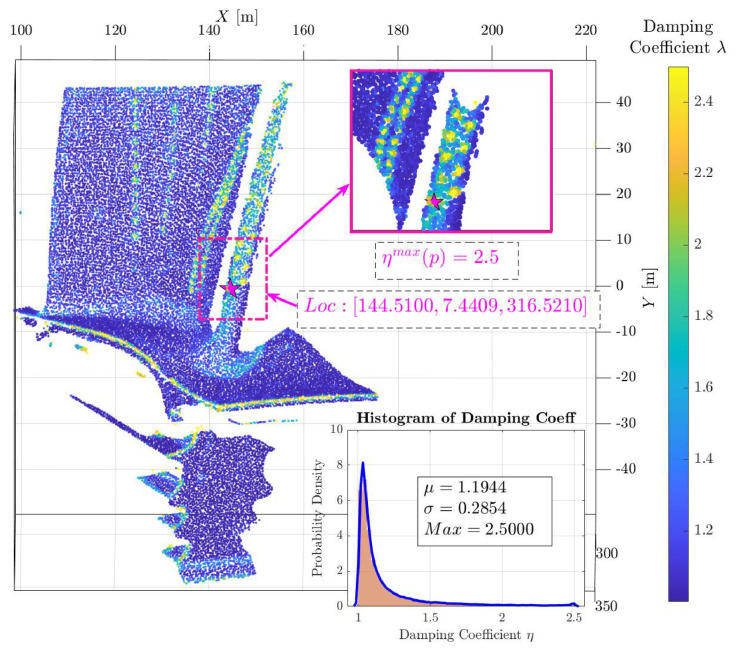
Anisotropic damping coefficient η(p) corresponding to individual $pop^{0}\left[1\right]$.

Using the formula $\eta \left(p\right)=1+\lambda_{damp}\;\cdot \;\left(1-\exp\left(-\frac{G{\left(p\right)}^{2}}{\sigma_{g}^{2}}\right)\right)$, the full-field damping coefficient map for the individual $pop^{0}\left[1\right]$ is calculated, as shown in Figure 6.

The mean value of the damping coefficient field is μ=1.1944, which is close to 1. This indicates that for the majority of the regions, a supervoxel boundary exchange is permitted provided that the energy function decreases following the exchange. Conversely, the damping coefficient corresponding to the point with the maximum multimodal gradient is $\eta^{max}\left(p\right)=2.5$. This implies that unless the exchange yields a significant advantage across all multimodal scales, the current assignment is maintained, thereby effectively preserving regions characterized by intense gradients.

Subsequently, for each individual in the population, the supervoxel reassignment algorithm based on local saliency is executed. The initial value of the global regularization parameter Λ is set to the median of the multi-objective weighted distances between each point and its nearest neighbor: $\Lambda =MED\left\{D_{4}^{X_{l}}\left(p_{i},Ne_{0}\left(i\right)\right)\left|p_{i}\in P\right.\right\}$, where $Ne_{0}\left(i\right)$ denotes the nearest neighbor of point $p_{i}$. The multi-objective weighted distance corresponding to individual $pop^{0}\left[1\right]$ is: $D_{4}^{X_{l}}\left(r_{k},p_{j}\right)=0.310\;\cdot \;D_{1}\left(r_{k},p_{j}\right)+0.257\;\cdot \;D_{2}\left(r_{k},p_{j}\right)+0.433\;\cdot \;D_{3}\left(r_{k},p_{j}\right)$.

At the initial stage of supervoxel reassignment, every point constitutes a supervoxel itself. The distances to nearest neighbors are calculated, and the distribution of the multi-objective weighted distances $D_{4}^{X_{l}}\left(p_{i},Ne_{0}\left(i\right)\right)$ between each point and its nearest neighbor is recorded. For individual $pop^{0}\left[1\right]$, the mean is $\mu \left(D_{4}^{X_{l}}\right)=5.2040$, the standard deviation is $\sigma \left(D_{4}^{X_{l}}\right)=6.5034$, and the maximum weighted distance is $\max\left(D_{4}^{X_{l}}\right)=101.4418$. Calculating the median yields the global regularization parameter Λ=3.4441. Next, based on the formula:

$$\Omega_{j}\left(\Lambda \right)=3.4441/1+0.257\;\cdot \;\sigma_{norm}\left(S_{j}\right)+0.433\;\cdot \;\sigma_{temp}\left(S_{j}\right)$$

the local adaptive penalty term for each supervoxel is calculated. Subsequently, utilizing the triangle inequality principle, it is determined whether each supervoxel should be merged. If the condition: $\Omega_{j}\left(\Lambda \right)>\left|Ra\left(j\right)\right|\;\cdot \;D_{4}^{X_{l}}\left(r_{j},r_{i}\right)$ is satisfied, the supervoxel is merged with the adjacent supervoxel.

**Figure 7:**
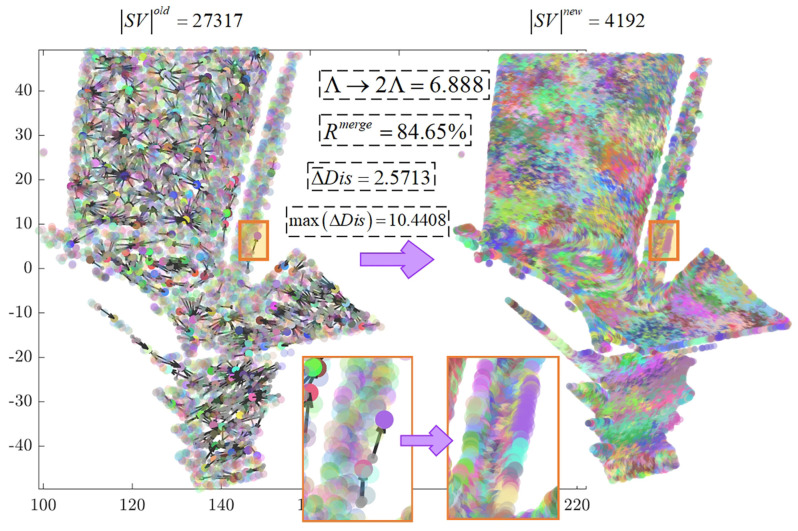
The first iteration of supervoxel reassignment.

The multiplication parameter is set to γ=2. In a bottom-up manner, adjacent supervoxels are rapidly merged based on the triangle inequality principle until the number of supervoxels is reduced to the expected value *K*. The process of the first round of merging is illustrated in Figure 7. The left image shows the initial state where all points form individual supervoxels, with a total count of ${\left|SV\right|}^{old}=27317$. After one round of reassignment, adjacent similar supervoxels are merged, resulting in a total supervoxel count of ${\left|SV\right|}^{new}=4192$. The reduction in supervoxels is 23,125, corresponding to a reduction rate of:

$$R^{merge}=\frac{{\left|SV\right|}^{old}-{\left|SV\right|}^{new}}{{\left|SV\right|}^{old}}=84.65\%$$

$\bar{\Delta }Dis$ represents the average physical distance moved by the merged center points to the new center points. The values $\bar{\Delta }Dis=2.5713$ and $\max\left(\Delta Dis\right)=10.4408$ indicate that merging occurs near adjacent supervoxels and does not span large spatial distances. The black arrows in the figure indicate the direction in which supervoxels are assimilated. After completing the neighborhood traversal for all supervoxels in this round, the global parameter is doubled, Λ→2Λ=6.888, to proceed to the next round of supervoxel reassignment.

**Figure 8:**
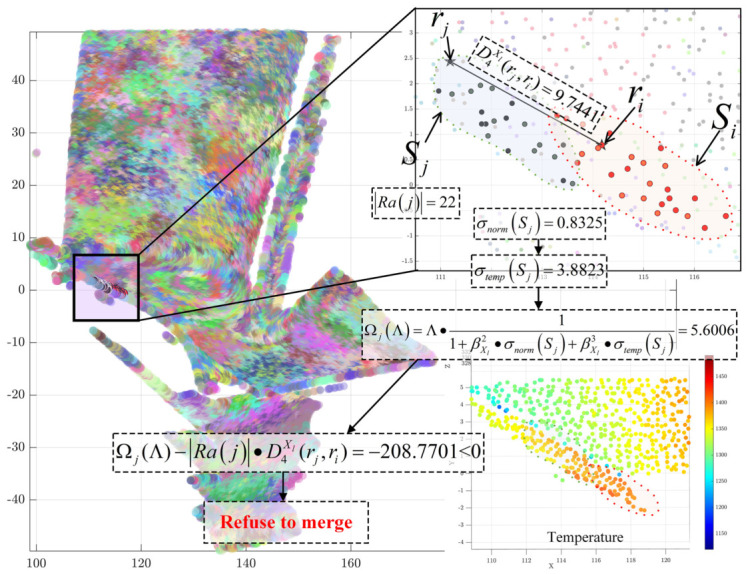
The supervoxel merging process based on local attribute saliency.

Figure 8 demonstrates the computational process for determining whether $S_{j}$ should be merged into $S_{i}$ during the reassignment phase for two adjacent supervoxels. To prevent the over-merging of salient multimodal features, the multi-objective weighted distance between the representative points of the two supervoxels is first calculated as: $D_{4}^{X_{l}}\left(r_{j},r_{i}\right)=0.310\;\cdot \;D_{1}\left(r_{j},r_{i}\right)+0.257\;\cdot \;D_{2}\left(r_{j},r_{i}\right)+0.433\;\cdot \;D_{3}\left(r_{j},r_{i}\right)=9.7441$.

The internal multimodal feature saliency of supervoxel $S_{j}$ is then computed, yielding a normal variation intensity of $\sigma_{norm}\left(S_{j}\right)=0.8325$ and a thermal variation intensity of $\sigma_{temp}\left(S_{j}\right)=3.8823$. The adaptive penalty term for merging $S_{j}$ is calculated as:

$$\Omega_{j}\left(\Lambda \right)=\frac{\Lambda }{1+0.257\;\cdot \;\sigma_{norm}\left(S_{j}\right)+0.433\;\cdot \;\sigma_{temp}\left(S_{j}\right)}=5.6006$$

At this stage, the global parameter has already been doubled to Λ=6.888. According to the triangle inequality principle, if the condition $\Omega_{j}\left(\Lambda \right)-\left|Ra\left(j\right)\right|\;\cdot \;D_{4}^{X_{l}}\left(r_{j},r_{i}\right)>0$ is met, the post-merge energy function is guaranteed to decrease. However, given that the total point count of $S_{j}$ is |Ra(j)|=22, the calculation yields: $\Omega_{j}\left(\Lambda \right)-\left|Ra\left(j\right)\right|\;\cdot \;D_{4}^{X_{l}}\left(r_{j},r_{i}\right)=-208.7701<0$.

This result indicates that, when accounting for multimodal feature saliency, supervoxel $S_{j}$ exhibits high feature complexity, likely representing a region with drastic thermal or morphological variations. Consequently, the algorithm rejects merging $S_{j}$ into $S_{i}$ and proceeds to evaluate the next neighboring supervoxel. This conclusion is visually corroborated by the point cloud temperature field image, where the distinct overall thermal colorations of supervoxels $S_{j}$ and $S_{i}$ suggest that they should not be fused.

**Figure 9:**
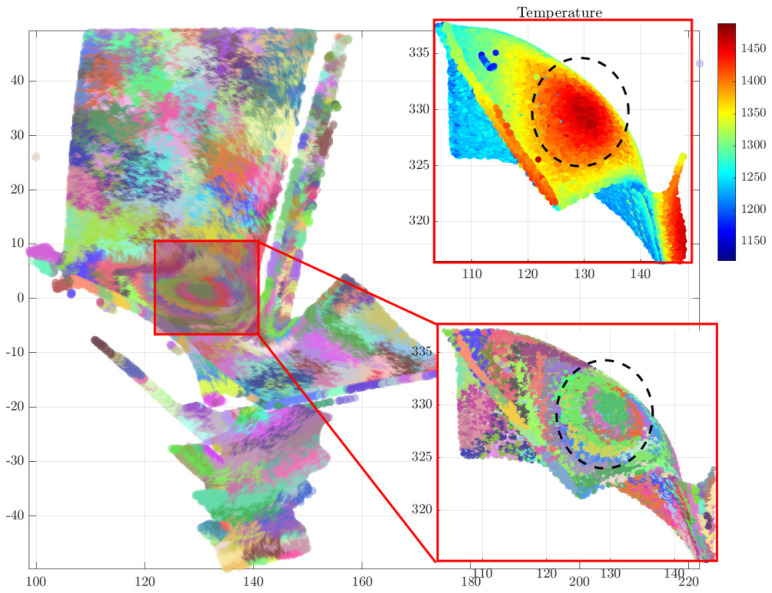
Result of the reassignment phase.

The final supervoxel reassignment result for individual $pop^{0}\left[1\right]$ is illustrated in Figure 9. As observed in the figure, the extracted supervoxels demonstrate robust boundary preservation, with their shapes closely conforming to the diffusion patterns of local thermal hotspots on the turbine blade. Furthermore, consistent with thermal propagation, regions corresponding to distinct temperature intervals are partitioned into separate supervoxels. This alignment allows the high-temperature point cloud segmentation to accurately reflect the heat transfer process, thereby enhancing segmentation precision. However, the boundary regions of the supervoxels currently exhibit a lack of clarity and jagged artifacts. Consequently, the gradient damping-based boundary exchange algorithm is required to further refine these boundaries, ensuring the preservation of physical features associated with authentic sharp edges or the margins of high-temperature heat sources.

To further enhance boundary consistency, an anisotropic supervoxel boundary exchange algorithm based on gradient damping is utilized for optimization. First, the boundary points of each supervoxel following the reassignment stage are identified. A point *p* is defined as a boundary point if its neighborhood contains any point with a different supervoxel label: ∃q∈N(p),s(q)≠s(p).

These points are pushed into a queue *Q*. For the reassignment result of individual $pop^{0}\left[1\right]$, the initial exchange queue size is |Q|= 26,573. The initial value of the multi-objective supervoxel segmentation energy function is $f_{4}\left(pop^{0}\left[1\right]|{\vec{\beta }}_{X_{l}},p^{*}\right)$ = 110,862.0920.

**Figure 10:**
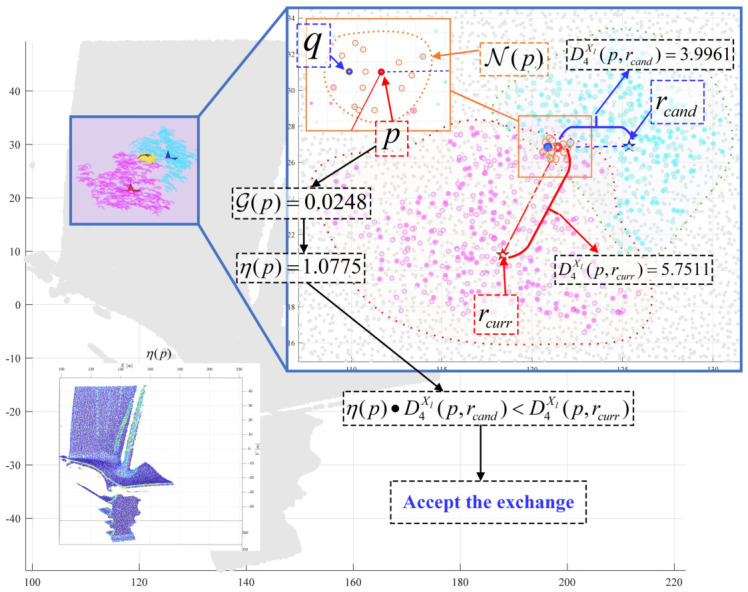
Supervoxel boundary exchange process.

Consider two supervoxels $S_{i}$ and $S_{j}$, as shown in Figure 10, with point counts Ra(i)=323 and Ra(j)=258, respectively. With a neighborhood size set to |N(p)|=15, the neighborhood of boundary point *p* (where $p\in S_{i}$) is searched, indicated by the yellow ring in the figure. In this neighborhood, a point $q\in S_{j}$ possesses a representative label inconsistent with that of *p*. The multimodal weighted distance between point *p* and its current representative is $D_{4}^{X_{l}}\left(p,r_{curr}\right)=5.7511$. If the label of *p* is reassigned to match that of *q* (i.e., changing to candidate representative $r_{cand}$), the new weighted distance becomes $D_{4}^{X_{l}}\left(p,r_{cand}\right)=3.9961$, suggesting a decrease in total energy.

To protect salient feature edges, the previously calculated gradient map G(p) and damping coefficient map η(p) are consulted to determine if *p* requires damping protection. For point *p*, the gradient is G(p)=0.0248 and the damping coefficient is η(p)=1.0775. Incorporating the damping factor, the comparison becomes: $\eta \left(p\right)\;\cdot \;D_{4}^{X_{l}}\left(p,r_{cand}\right)=4.3057<D_{4}^{X_{l}}\left(p,r_{curr}\right)=5.7511$. This indicates that the feature intensity at this point is relatively low. Reassigning *p* to $r_{cand}$ successfully lowers the energy function; thus, the representative label of *p* is updated to $r_{cand}$, and *q* is added to the end of the queue. The energy function before the exchange was $f_{4}^{before}=$ 110,860.3370, and after the exchange, it becomes $f_{4}^{after}=$ 110,858.5821. This specific label exchange contributes an energy reduction of $\Delta f_{4}=-$1.7549.

**Figure 11:**
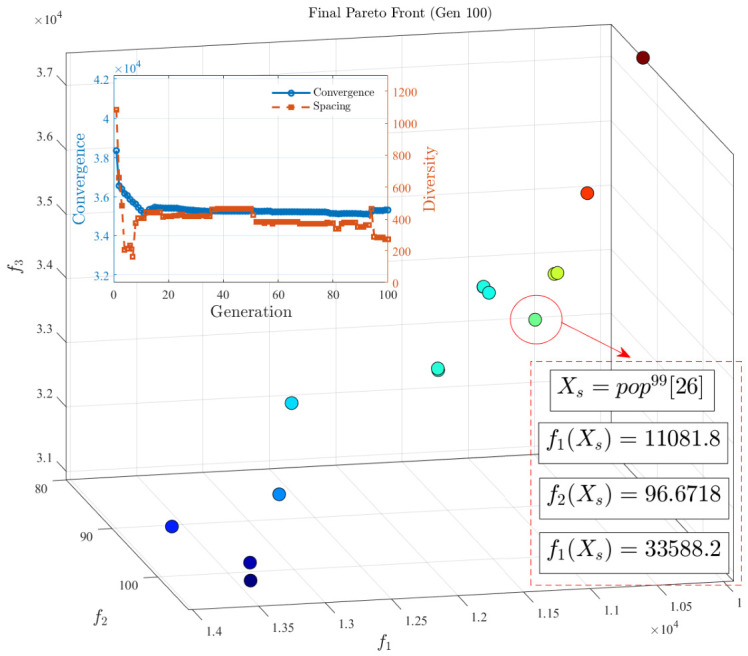
Final pareto front.

After executing the supervoxel reassignment and boundary exchange algorithms for each individual in the population, the corresponding supervoxel allocation schemes are obtained. The decomposed objective function value is calculated as $g\left(X|\vec{\beta },p^{*}\right)=d_{1}\left(X\right)+\alpha d_{2}\left(X\right)$. Dominance is then evaluated, and non-dominated solutions are retained. Through continuous population evolution, the final Pareto Front is generated, as shown in Figure 11. The spacing metric (distribution index) is calculated as $S=\sqrt{\frac{1}{n-1}\sum_{i=1}^{n}{\left(d_{i}-\bar{d}\right)}^{2}}$, where *n* is the number of solutions in the Pareto set, $d_{i}$ is the distance from the *i*-th solution to its nearest neighbor in the objective space, and $\bar{d}$ is the average value of $d_{i}$. The convergence metric is defined as the average distance from the solutions to the origin. For both metrics, a smaller value indicates superior performance. As the iterations progressed, the distribution index decreased from 1083.2606 to a final value of 270.874, while the convergence index decreased from 38,354.2054 to 35,332.1037. This demonstrates that the penalty-based decomposition method achieves a robust balance between convergence and diversity. A compromise solution, $X_{s}=pop^{99}\left[26\right]$, was selected. Its corresponding values for the three objective functions are $f_{1}\left(X_{s}\right)=$ 11,081.8, $f_{2}\left(X_{s}\right)=$ 96.6718, and $f_{3}\left(X_{s}\right)=$ 33,588.2.

**Figure 12:**
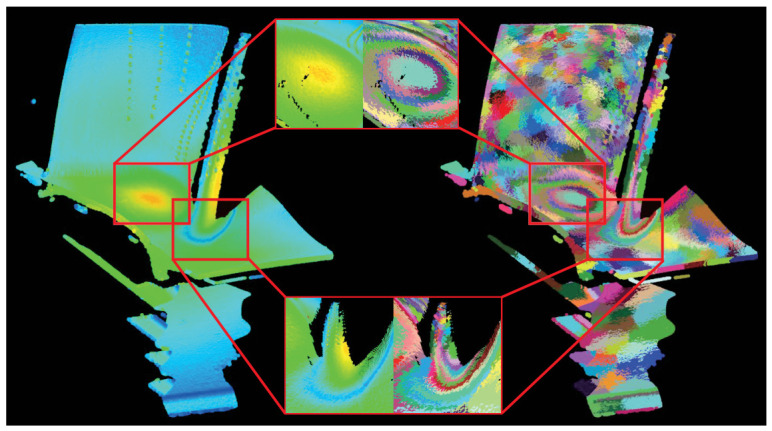
Original scale multi-objective supervoxel point cloud segmentation result.

The multi-objective optimal weight ratio corresponding to the compromise solution $X_{s}$ is found to be ${\left[0.337,0.501,0.161\right]}^{T}$. These optimal parameters are passed back to the original point cloud scale to execute the final segmentation. Based on this weight distribution, a multi-objective supervoxel segmentation objective function is constructed for the full-scale point cloud, and the final segmentation is achieved through the reassignment and boundary exchange algorithms. The results are illustrated in Figure 12. As shown, the multi-objective supervoxel segmentation method successfully incorporates multimodal information into a comprehensive partitioning process. The resulting supervoxels not only provide a fine-grained segmentation of the local heat source diffusion patterns but also precisely isolate areas with significant normal variations, such as the arrayed cooling holes. This demonstrates high-precision segmentation for multimodal point cloud data.

**Figure 13:**
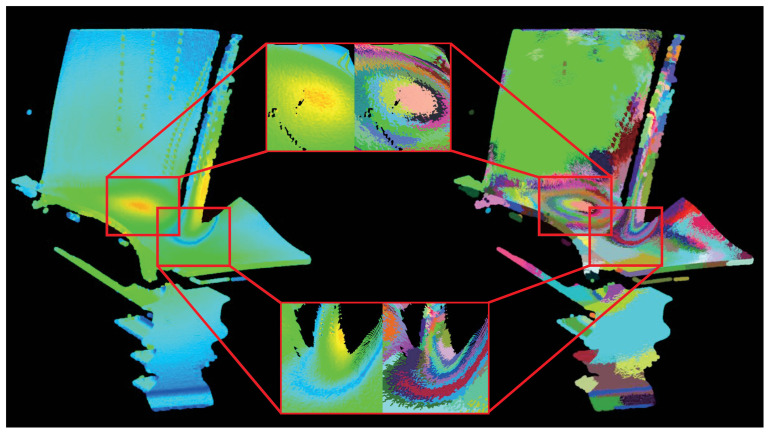
Original scale supervoxel region merging results.

After performing multi-objective supervoxel segmentation at the original scale, a region-merging process is required to obtain the final point cloud segmentation results. A region-growing-based merging algorithm is established using information from the Pareto-optimal compromise solution. The merging threshold is set to $Thr_{md}=1.5$, and the planar residual threshold is set to φ=0.1. The similarity $M(R_{i},R_{j})$ between supervoxels is calculated to consolidate similar regions. The results are illustrated in Figure 13. Before merging, the total number of supervoxels was 377; through the merging operation, 154 supervoxels were consolidated. It is evident that the region-growing merging method effectively combines similar supervoxels while preserving significant regions of temperature fluctuations and normal variations.

### 4.1. Algorithm Performance Comparison

#### 4.1.1. Performance Comparison of Supervoxel-Level Segmentation Algorithms

To further demonstrate the advantages of the proposed multi-object supervoxel segmentation method in addressing multimodal high-temperature point cloud segmentation, we compared our approach against mainstream segmentation methods. To ensure a rigorous and fair evaluation, we categorize the comparison into two distinct levels based on the granularity of segmentation: supervoxel-level evaluation and object-level evaluation.

The baseline algorithms for the supervoxel segmentation comparison include the traditional voxel-based VCCS method [14], the point-based VCCS_KNN [16] method, the octree-based method [26], the Linear Spectral Clustering (LSC) method [27], the distance-based SLIC method [28], and the TBPS method [16]. Because supervoxel algorithms are inherently designed for over-segmentation, utilizing region-level metrics such as Recall or Intersection over Union (IoU) for their evaluation is mathematically flawed. The performance metrics selected for supervoxel segmentation include standard segmentation metrics, Boundary Recall (BR_G) [14,16], Under-segmentation Error (UE) [28], and Achievable Segmentation Accuracy (ASA), as well as supervoxel-specific metrics. Specifically, the Explained Variance of Geometry ($EV_{geom}$) [17,18] measures the retention rate of the original geometric information following supervoxelization. A higher value indicates superior reconstruction of the object’s surface shape:

$$EV_{geom}=\frac{\sigma_{total\text{\_}geom}^{2}-\sum_{i}w_{i}\;\cdot \;\sigma_{i\text{\_}geom}^{2}}{\sigma_{total\text{\_}geom}^{2}}$$

where $\sigma_{total\text{\_}geom}^{2}$ represents the geometric variance of the entire point cloud, $\sigma_{i\text{\_}geom}^{2}$ is the geometric variance within the *i*-th supervoxel, and $w_{i}$ is the weight (number of points in the supervoxel divided by the total points). An $EV_{geom}$ closer to 1 indicates the supervoxels perfectly fit the object’s surface shape.

Geometric compactness $C_{geom}$ measures the regularity of the generated supervoxel shapes:

$$C_{geom}=\frac{V_{sv}}{V_{aabb}}$$

where the actual volume of the supervoxel is divided by the volume of its Axis-Aligned Bounding Box (AABB). A larger value indicates more regular supervoxels.

Furthermore, to evaluate the performance in the thermal dimension, we extend these metrics to include Weighted Temperature Variance (WTV), Temperature Explained Variance ($EV_{temp}$), Intra-region Temperature Homogeneity (ITH), and Temperature Boundary Recall ($BR_{T}$), inspired by their geometric counterparts:

WTV measures the discrete loss of the global segmentation results in the temperature dimension:

$$WTV=\frac{1}{N}\sum_{i=1}^{K}\sum_{p\in S_{i}}{\left(T_{p}-{\bar{T}}_{S_{i}}\right)}^{2}$$

where *N* is the total number of points, *K* is the number of supervoxels, $S_{i}$ is the *i*-th supervoxel, $T_{p}$ is the temperature of point *p*, and ${\bar{T}}_{S_{i}}$ is the average temperature of that supervoxel. A lower WTV indicates higher thermal consistency within supervoxels, signifying that points with large temperature differences were not forcibly aggregated.

For high-temperature point cloud segmentation, each segment should ideally have a uniform internal temperature. ITH is introduced as:

$$ITH=\frac{1}{K}\sum_{k=1}^{K}\sqrt{\frac{1}{|S_{k}|}\sum_{p\in S_{k}}{\left(T_{p}-{\bar{T}}_{k}\right)}^{2}}.$$

This represents the average of the internal temperature standard deviations across all supervoxels; a lower value is preferable.

Geometric Boundary Recall ($BR_{G}$) measures the proportion of original geometric edges points successfully partitioned by supervoxel boundaries, indicating boundary preservation performance. Similarly, we introduce Temperature Boundary Recall ($BR_{T}$) to measure the proportion of temperature mutation zones or high-gradient points successfully covered by the supervoxel boundaries:

$$BR_{T}=\frac{|B_{sv}\cap B_{T}^{gt}|}{|B_{T}^{gt}|}.$$

Similar to $EV_{geom}$, we extend this to the thermal attribute. $EV_{temp}$ measures the information retention rate of the original temperature field:

$$EV_{temp}=\frac{\sigma_{total\text{\_}temp}^{2}-\sum_{i}w_{i}\;\cdot \;\sigma_{i\text{\_}temp}^{2}}{\sigma_{total\text{\_}temp}^{2}}.$$

**Figure 14:**
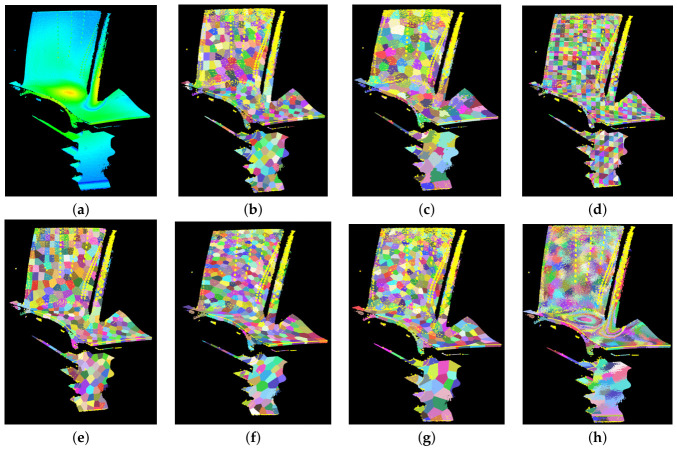
Comparison of supervoxel segmentation algorithms: (**a**) High-temperature point cloud; (**b**) VCCS result; (**c**) VCCS_KNN result; (**d**) Octree result; (**e**) SLIC result; (**f**) LSC result; (**g**) TBPS result; (**h**) Proposed multi-objective supervoxel results.

Comparative experiments on supervoxel segmentation were conducted using the high-temperature turbine blade data mentioned previously. The segmentation results are illustrated in Figure 14, and the calculated quantitative metrics are presented in Table 1. The yellow points in Figure 14 represent the top 5% of salient points ranked by temperature and normal gradients. The results indicate that our proposed method demonstrates superior boundary adherence and segmentation accuracy. As shown in the table, our method achieved the highest ASA of 96.27% and the lowest UE of 4.96%. More importantly, our method attained a BR_G of 37.5%. This demonstrates that the generated supervoxels successfully and strictly adhere to the true physical boundaries without arbitrary overstepping.

A common challenge in multimodal fusion segmentation is the potential degradation of geometric regularity. As shown in Table 1, our method’s performance on supervoxel-specific metrics is not particularly striking. This is primarily because temperature fields and spatial geometric features are often not perfectly aligned; local thermal hotspots may be distributed across geometrically flat regions. Traditional supervoxel-specific metrics encourage the formation of highly regular spherical or cubic shapes. In reality, due to the lack of thermal awareness, these rigid shapes inevitably disrupt or blur the actual thermal boundaries.

Under these circumstances, our algorithm relaxes rigid spatial constraints, allowing supervoxels to deform in order to prioritize genuine heat transfer boundaries. Nevertheless, as indicated by the supervoxel-specific metrics in the table, although our segmentation method is deeply guided by thermal features, the generated supervoxels remain highly competitive with purely geometry-driven baseline methods, such as Octree and VCCS, in terms of geometric preservation and compactness, maintaining an $EV_{geom}$ of 99.28%. Through a minor compromise in pure geometric rigidity, our method achieves a significant leap in thermal awareness. It attains the highest thermal explained variance ($EV_{temp}=94.04\%$) and the lowest WTV (WTV=202.23). Compared to VCCS, the WTV is reduced by approximately 65%. Crucially, our thermal boundary recall reaches 36.53%. This demonstrates that our method acquires robust thermal segmentation capabilities without sacrificing critical geometric performance, successfully realizing the fusion of geometry and temperature.

#### 4.1.2. Performance Comparison of Object-Level Segmentation Algorithms

For object-level segmentation algorithms, the compared baseline algorithms include classical segmentation methods, such as Euclidean Clustering (EC) [9], Region Growing (RG) [29], and normal-based segmentation algorithms [30]. Additionally, supervoxel-based segmentation and merging algorithms are included, encompassing the graph-based LCCP algorithm [31], Octree + RG, SLIC + RG, and LSC + RG. In addition to $BR_{G}$, UE, and ASA, the standard metrics for performance evaluation also encompass common point cloud segmentation metrics [28,32]: Variation of Information (VI), Precision, Recall, F1-score, and Intersection over Union (IoU). Concurrently, Boundary Thermal Contrast (BTC), is introduced to measure the thermal response flow tracking capability of the segmented regions. For an optimal segmentation, the temperature gradient at the boundaries should be significantly greater than the temperature gradient within the internal regions. Therefore, the formula for calculating BTC is as follows:

$$BTC=\frac{\bar{\nabla }T_{boundary}}{\bar{\nabla }T_{internal}}$$

A higher BTC metric indicates that the segmentation boundaries can accurately delineate locations with abrupt temperature changes, whereas a lower BTC value suggests a failure to effectively partition regions with significant temperature variations.

**Figure 15:**
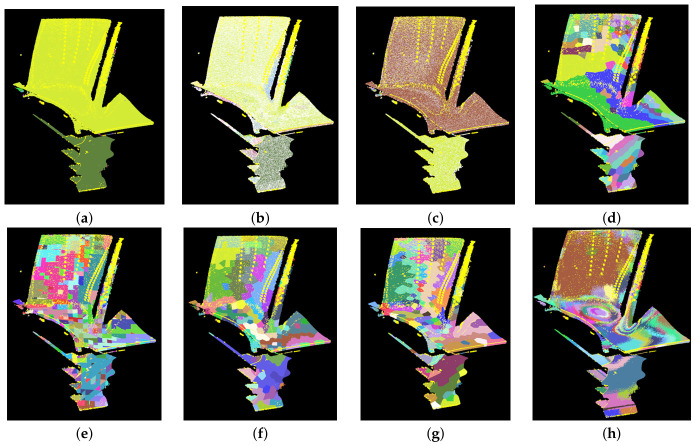
Comparison results of object-level segmentation algorithms: (**a**) EC segmentation result; (**b**) RG segmentation result; (**c**) Normal-based segmentation result; (**d**) LCCP (Graph) segmentation result; (**e**) Octree + RG segmentation result; (**f**) SLIC + RG segmentation result; (**g**) LSC + RG segmentation result; (**h**) Segmentation result of the proposed method.

The comparative results and performance of region-level object segmentation are shown in Figure 15 and Table 2, respectively. As can be seen from the table, the Recall and IoU of global clustering methods such as EC are mathematically inflated, reaching 99.99% and 85.52%, respectively. However, their corresponding boundary recall metrics, $BR_{G}$ and $BR_{T}$, are extremely low, and the WTV is as high as 3334.07. This indicates a catastrophic under-segmentation failure; the algorithm essentially merged the entire scene into a single massive cluster, rendering the high recall physically meaningless, which can also be observed in the segmentation result figures. Conversely, traditional region growing captures boundaries well but suffers from severe spatial fragmentation, as evidenced by its extremely low Achievable Segmentation Accuracy (ASA = 47.04%). These extreme cases demonstrate that direct point-level clustering is insufficient for point clouds of turbine blades with complex curved surfaces.

Within the two-stage segmentation framework, our proposed method exhibits state-of-the-art robustness. It achieves the highest precision of 97.94% and the highest ASA of 95.96%. Among the compared methods, the classical graph-based LCCP suffers from a higher under-segmentation error because its purely geometry-based concavity rules blindly cross thermal boundaries. In contrast, our method limits the UE to 7.45%. This proves that our fusion-based merging strategy effectively prevents different physical or thermal regions from interpenetrating.

Our method is most prominently validated by thermal-specific metrics. Our full fusion pipeline achieves the highest thermal explained variance ($EV_{temp}=89.32\%$) and the lowest WTV among all two-stage methods. Notably, our method achieves the highest Boundary Thermal Contrast (BTC=1.33). A BTC value significantly greater than 1.0 explicitly indicates that our final segmentation boundaries are strictly aligned with drastic temperature gradients, maximizing inter-regional variance while maintaining high intra-regional thermal uniformity.

#### 4.1.3. Performance Comparison of Baseline Methods Adapted for Thermal Features

When comparing our proposed framework with traditional supervoxel baselines, a direct comparison of thermal segmentation performance is intrinsically unfair. Most conventional methods can only process 3D spatial or RGB-D data and rely strictly on spatial or color distance metrics, meaning they cannot directly incorporate temperature features. To establish a fair basis for comparison, we adopted a straightforward approach: mapping the one-dimensional temperature scalars into a three-dimensional pseudo-color space, thereby allowing the baseline algorithms to accept thermal data.

We acknowledge that this mapping strategy is not perfect. Due to the highly nonlinear nature of pseudo-color mapping, it inherently distorts the distance metrics. Specifically, a constant temperature variation (ΔT) can result in significantly different Euclidean color distances depending on its position within the color map. Although this nonlinear transformation inevitably compromises the clustering accuracy of the baseline methods, we consider it a reasonable and acceptable workaround to facilitate a fair benchmark evaluation for these traditional algorithms. Conversely, our proposed method directly and natively processes thermodynamic features. This native processing capability underscoring its critical importance and intrinsic superiority in preserving authentic thermal gradients for precise multi-modal segmentation.

The specific implementations for adapting the baselines to temperature features are as follows: For algorithms relying on the Point Cloud Library (PCL) [33], such as Region Growing and VCCS, which provide interfaces for RGB inputs, we converted the normalized temperature values into the Hue channel of the HSV color space and subsequently mapped them to RGB to generate pseudo-color thermal maps. For the LSC algorithm, we implemented LSC (temp) by explicitly projecting the 3D spatial coordinates and temperature values into an 8-dimensional feature space to perform kernelized K-means clustering. For the TBPS method, the mapped RGB values were utilized as the distance metric component, effectively rendering it a single-objective segmentation method with fixed weights. To evaluate the final object-level segmentation performance, these generated supervoxels were subsequently merged using either Region Growing or LCCP.

**Figure 16:**
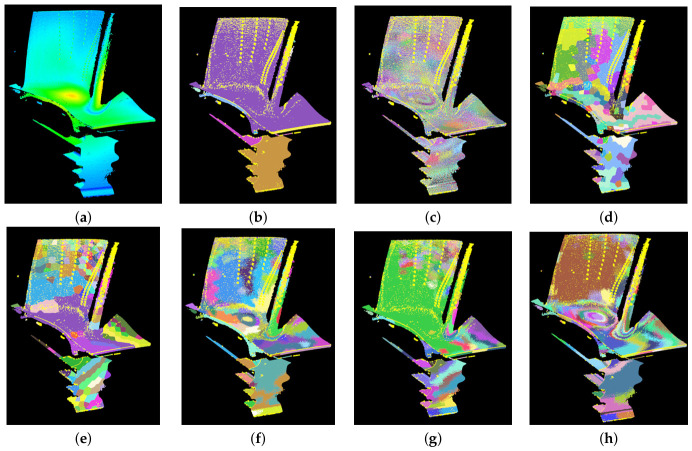
Comparison of segmentation performance of baseline algorithm adapted for thermal features: (**a**) High temperature point cloud; (**b**) RG (color) result; (**c**) LSC (temp) result; (**d**) VCCS (color) + RG result; (**e**) VCCS (color) + LCCP result; (**f**) TBPS (color) + RG result; (**g**) TBPS (color) + LCCP result; (**h**) Segmentation result of the proposed method.

The comparative results and performance data are presented in Figure 16 and Table 3. As can be observed, directly injecting temperature features into point-level clustering algorithms often yields suboptimal results. For instance, although RG (color) exhibits the highest Recall and IoU, its significantly low boundary recall and extremely high WTV reveal severe under-segmentation. Relying on simple color thresholds causes the algorithm to merge thermally inconsistent regions. Conversely, LSC (temp) achieves a high boundary recall due to its tendency to generate highly fragmented and irregular patches. However, this fragmentation is exposed by its high under-segmentation error and poor thermal consistency.

Extending supervised voxel algorithms, such as VCCS and TBPS, to include color features can enhance local thermal awareness, but the subsequent merging phase often disrupts this consistency. When utilizing LCCP to merge VCCS (color) and TBPS (color), the WTV increases significantly. This is because LCCP relies strictly on geometric concavity and convexity rules for merging, completely ignoring the thermal attributes of the supervised voxels. Even when using RG for merging, the thermal consistency remains inferior to our method. This indicates that treating temperature merely as an additional distance component with a fixed weight is insufficient for handling complex multi-modal boundaries.

Our proposed method significantly outperforms all temperature-extended baseline methods by dynamically and intrinsically balancing geometric and thermal features. Our method achieves the highest Precision and ASA, ensuring that the segmentation does not erroneously cross genuine physical boundaries. Most importantly, our method demonstrates a distinct advantage in thermal-specific metrics. It achieves the highest thermal explained variance and the lowest WTV. Compared to the best-performing extended baseline method, our approach reduces the WTV by an additional 28.8%. These results explicitly prove that our specific fusion strategy is superior to the approach of simply concatenating thermal features into existing algorithms.

## 5. Discussion

### 5.1. Parameter Sensitivity Analysis

The selection of parameters is of paramount importance for balancing the sensitivity to scalar field gradients and the capability to preserve geometric structural integrity. To elucidate the selection mechanism of the damping coefficient parameters and validate their generalization capability, we conducted a comprehensive sensitivity analysis on the gradient sensitivity parameter $\sigma_{g}$ and the maximum damping gain $\lambda_{damp}$.

**Figure 17:**
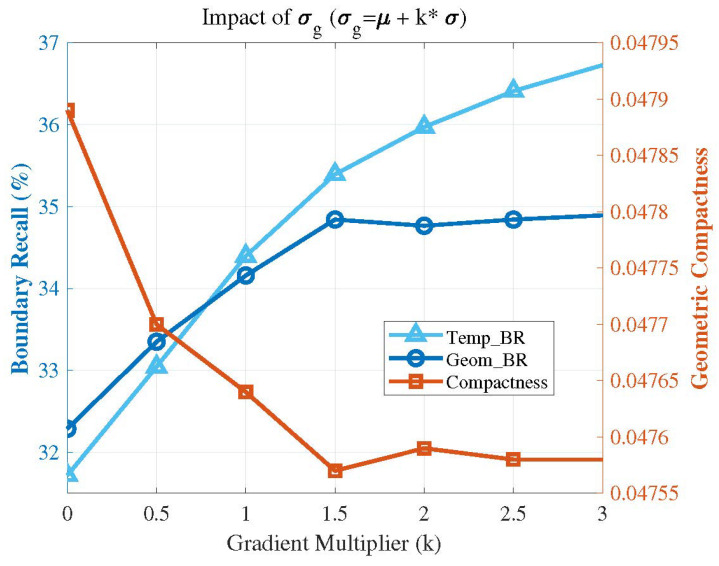
Performance impact of parameter $\sigma_{g}$.

Figure 17 illustrates the performance impact of the parameter $\sigma_{g}$. As described in the experimental implementation details, the value of the parameter $\sigma_{g}$ is derived from the statistical distribution of the gradient map, and is formulated as $\sigma_{g}=\mu +k\;\cdot \;\sigma$. Therefore, we conducted a performance validation by incrementally increasing the multiplier *k* from 0 to 3. As can be observed from the figure, the thermal boundary recall (Temp_BR) continuously rises as the parameter σ_g increases. However, excessive boundary sensitivity leads to severe over-segmentation, which steadily degrades the geometric compactness of the resulting supervoxels, compromising their regularity and, consequently, the overall segmentation accuracy. By setting k=2, the algorithm adaptively identifies the top 5% of statistically significant gradients as genuine multi-modal boundaries. This configuration preserves well-shaped and regular spatial structures while achieving substantial temperature boundary preservation performance. Furthermore, this adaptive approach based on gradient statistics empowers the algorithm to dynamically accommodate the diverse temperature scales and sensor noise levels inherent in different datasets.

**Figure 18:**
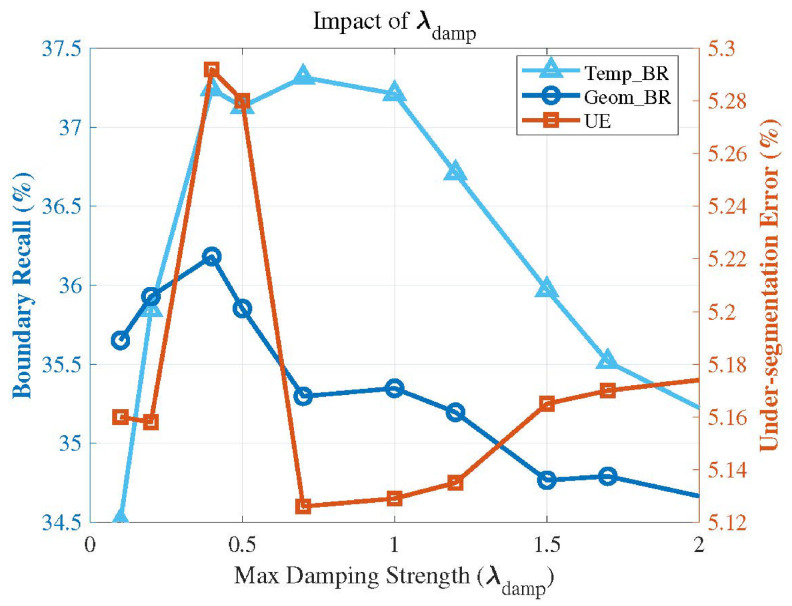
Performance impact of parameter $\lambda_{damp}$.

The parameter $\lambda_{damp}$ controls the maximum penalty imposed when crossing detected boundaries, which serves as the core mechanism for preventing over-smoothing. To demonstrate that our method strictly avoids over-smoothing, we evaluated the trade-off between boundary recall and under-segmentation error (UE). As can be observed from Figure 18, although a lower damping intensity $\lambda_{damp}$, such as around 0.4, yields a higher theoretical boundary recall, it simultaneously causes a surge in the under-segmentation error. This weaker penalty fails to strictly constrain the region growing process, causing supervoxels to occasionally overflow physical boundaries, thereby resulting in a pronounced spike in UE.

To fundamentally eliminate over-smoothing, we adopted a conservative and strong damping setting of $\lambda_{damp}=1.5$ to enforce a strict penalty at multi-modal boundaries. While this conservative setting slightly restricts the absolute maximum boundary recall, it drastically reduces the UE, successfully preventing the intermixing of distinct physical or thermal regions. Ultimately, the broad and stable performance plateaus observed for both parameters in the two figures validate the robustness of our framework, confirming its ability to deliver state-of-the-art segmentation capabilities across diverse scenarios with a highly reliable default configuration.

Additionally, beyond the initial generation of supervoxels, the region growing merging step relies on a distance threshold ($Thr_{md}$) to determine cluster similarity. To validate the robustness of the selected threshold $Thr_{md}=1.5$, we conducted a comprehensive sensitivity analysis, exploring the trade-off between over-segmentation and under-segmentation over a range from 0.5 to 3.0.

**Figure 19:**
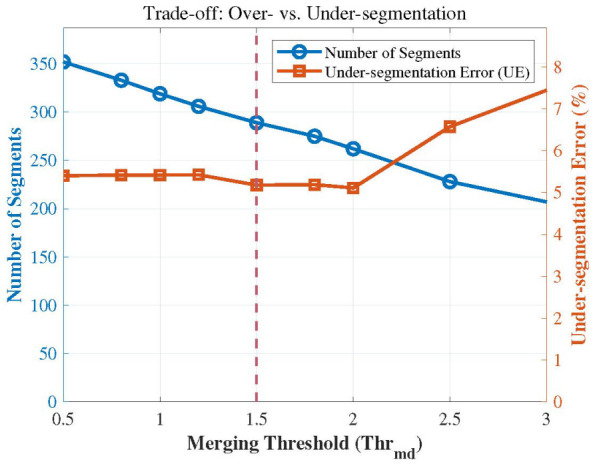
Influence of Region Growth Threshold $Thr_{md}$ on Merging Performance and UE.

Figure 19 illustrates the impact of $Thr_{md}$ on structural integrity. As can be seen from the figure, when the threshold is excessively stringent ($Thr_{md}<1.0$), the algorithm rejects meaningful merges, resulting in over-segmentation. Consequently, a large number of supervoxel patches remain, with the total number of segmented patches exceeding 300. As $Thr_{md}$ increases, adjacent clusters are successfully merged, and the total number of regions steadily decreases, thereby producing more meaningful object-level clustering. However, with an overly lenient threshold, such as $Thr_{md}>2.0$, structurally distinct regions are erroneously merged, causing the under-segmentation error (UE) to climb sharply.

**Figure 20:**
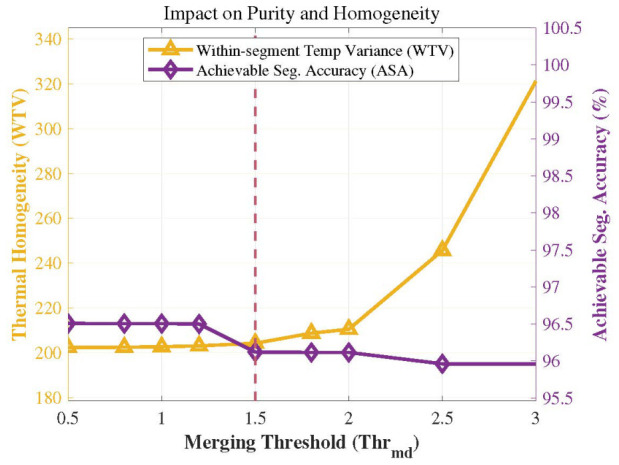
Influence of Region Growth Threshold $Thr_{md}$ on Segmentation Performance.

Figure 20 demonstrates the impact of the threshold on segmentation performance. As observed from the figure, the WTV metric maintains a low and stable value up to $Thr_{md}=1.5$. When the threshold $Thr_{md}$ gradually increases beyond 2.0, the WTV metric exhibits an exponential surge. This indicates that thermodynamic microstructures with significant temperature differences, such as local hotspots and the background, are incorrectly fused together. Concurrently, the achievable segmentation accuracy (ASA) begins to decline. In conclusion, setting the supervoxel merging threshold to 1.5 is a rational configuration. It maximizes the merging of structurally similar regions while circumventing an explosive increase in thermodynamic variance and under-segmentation error.

### 5.2. Down-Sampling Impact Analysis

When processing dense multi-modal point clouds, a common concern is that down-sampling might discard critical thermodynamic microstructures relevant to defect detection. To resolve the conflict between computational efficiency and the preservation of microscopic defects, our framework adopts an architecture comprising optimization at the down-sampled layer and execution at the full-resolution layer. Down-sampling is exclusively employed to accelerate the multi-objective evolutionary algorithm during the search phase for optimal multi-modal weight parameters. Once the optimal parameters are learned, they are applied to the full-resolution original point cloud for the final supervoxel segmentation.

Utilizing a down-sampled point cloud as an optimization surrogate is mathematically feasible and physically sound due to three key thermodynamic characteristics: (a) Low-frequency nature of thermal fields: Governed by Fourier’s law of heat conduction, thermal anomalies diffuse into macroscopic footprints, allowing the voxelized surrogate to accurately retain essential thermodynamic gradients despite the down-sampling of high-frequency geometric details; (b) Scale invariance of objective weights: The physical relationship between geometric and thermal features depends on inherent material properties, making the optimized relative fusion weights scale-invariant. Projecting these learned weights back onto the full-resolution data for final segmentation ensures zero loss of micro-level structures; (c) Spatial low-pass filtering: Down-sampling inherently acts as a spatial low-pass filter against high-frequency sensor noise and local emissivity variations, enhancing the signal-to-noise ratio (SNR) of authentic thermodynamic trends and guiding the optimizer toward more robust physical parameters.

**Figure 21:**
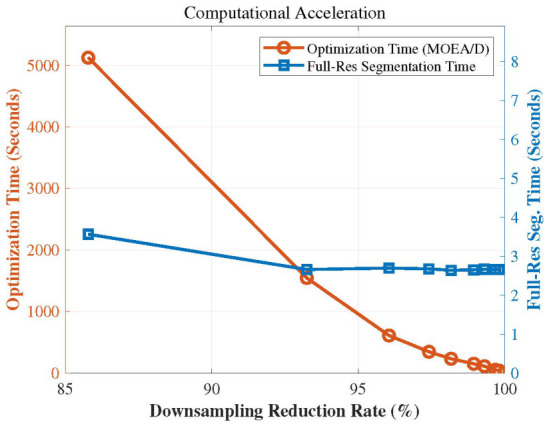
Variation of computational efficiency with sampling rate.

To further demonstrate the necessity of down-sampling and its capability to preserve original fine structures, we conducted a comprehensive study on the segmentation performance and computational efficiency across various sampling parameters. The down-sampling scale parameter was varied from 0.2 to 2.0. The variation of computational efficiency with respect to the sampling rate across different down-sampling scales is illustrated in Figure 21. As depicted in the figure, due to its iterative evolutionary nature, directly executing MOEA/D on dense point clouds inevitably incurs a severe computational bottleneck. However, by performing the parameter search on the down-sampled surrogate model, the optimization time is drastically reduced by 5087.58 s, achieving a 135.7-fold acceleration. Concurrently, the actual full-resolution segmentation time remains completely unaffected by the optimization phase, maintaining a rapid and exceptionally stable execution efficiency of 2.76±0.30 s. This demonstrates that our strategy successfully isolates and accelerates the specific computational bottleneck.

**Figure 22:**
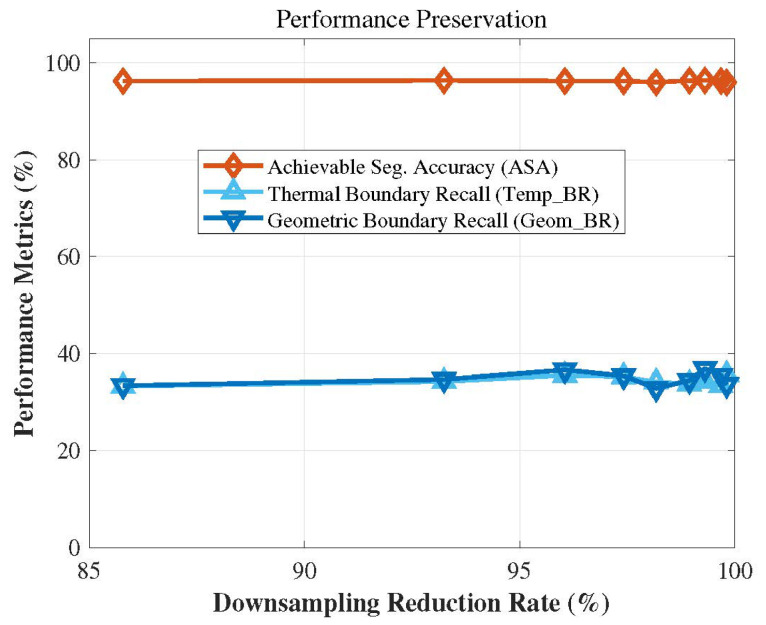
Variation of segmentation performance with sampling rate.

Figure 22 illustrates the impact of different sampling rates on the supervoxel segmentation performance of the original full-resolution point cloud. As observed from the figure, across a substantial range of down-sampling rates, the trajectories of the core metrics remain essentially flat horizontal lines. Quantitatively, the ASA metric is strictly maintained at 96.24±0.15%, while the thermodynamic boundary recall (Temp_BR) fluctuates within the range of 33.28% to 35.65%, and the geometric boundary recall (Geom_BR) is robustly preserved within the 32.82% to 36.89% interval. Compared to the traditional supervoxel methods evaluated in the previous performance comparison section, our method still retains a distinct advantage. The minor standard deviations among these metric parameters indicate that the proposed bi-level framework achieves massive computational acceleration while concurrently preserving stable segmentation performance and local fine structures.

### 5.3. Limitations and Future Work

Although the proposed segmentation framework integrating 3D geometric and thermal point clouds demonstrates exceptional performance in balancing computational efficiency and the preservation of thermodynamic microstructures, we acknowledge that the current experimental scope possesses certain limitations. Due to the inherent difficulties in acquiring spatially aligned 3D-temperature multi-modal point clouds in extreme industrial environments, there is a scarcity of publicly available benchmark datasets. Although our algorithmic architecture incorporates a multi-objective evolutionary method for the dynamic learning of multi-modal weight ratios, enabling the autonomous adaptation of fusion weights to novel structures, its physical generalization capability to other complex components (such as combustion chambers or nozzles) remains to be empirically validated. Future research efforts will focus on the following directions:

1. Expanding the industrial high-temperature 3D-thermal point cloud dataset by introducing more complex components (e.g., combustion chambers and nozzles) to validate the generalizability of the dynamic parameter optimization architecture across a broader range of high-temperature components.
2. Extending the representation of temperature features within traditional supervoxel algorithms. As previously noted, nonlinear pseudo-color mapping inevitably distorts the corresponding distance metrics. It is necessary to fundamentally upgrade the underlying mathematical principles of these classical baseline methods to natively support the direct processing of raw scalar fields, thereby expanding their multi-physics processing capabilities.
3. Enhancing online and real-time capabilities. The nature of evolutionary optimization inherently requires iterative computation, which may pose challenges for online inspection. Future research will explore learning-based parameter prediction to infer optimal fusion weights directly from topological features, thereby substantially improving real-time performance.

## 6. Conclusions

This paper presents a systematic framework for thermo-geometric point cloud supervoxel segmentation, specifically designed to address the critical challenge of integrating heterogeneous outputs from spatial 3D scanners and thermal imaging sensors. By employing a decomposition-based multi-objective evolutionary algorithm, the cross-modal segmentation problem is vectorially decomposed, enabling weight learning that ensures an objective balance between spatial compactness, normal consistency, and thermal homogeneity. The proposed anisotropic supervoxel generation algorithm, grounded in a gradient damping mechanism, applies adaptive energy penalties based on local sensor signal saliency. This allows for the simultaneous capture of subtle thermal anomalies recorded by thermometry sensors and sharp geometric edges captured by spatial sensors. Finally, supervoxels are merged into a complete segmentation based on the derived optimal multi-sensor fusion weights. Experimental results demonstrate that our method achieves superior segmentation performance, particularly in terms of cross-modal gradient boundary preservation. The framework is capable of performing refined segmentation on industrial high-temperature point clouds and accurately extracting localized thermal anomaly regions.

## Figures and Tables

**Table 1:** Supervoxel segmentation performance comparison.

**Method**	**Standard Segmentation Metrics**			**Supervoxel-Related Metrics**	
	**BR_G (↑)**	**ASA (↑)**	**UE (↓)**	**EV_geom (↑)**	**Geom Compactness (↑)**
VCCS	5.31	94.72	11.42	99.71	0.02
VCCS_KNN	15.63	93.88	15.08	95.58	0.02
Octree	10.77	95.58	9.02	**99.83**	0.07
SLIC	4.35	94.7	11.53	99.7	0.02
LSC	5.01	94.08	12.87	98.07	0.02
TBPS	6.09	95.88	6.72	99.7	**0.08**
Ours	**37.5**	**96.27**	**4.96**	99.28	0.03
**Method**	**Thermal-Specific Metrics**				
	**EV_temp (↑)**	**WTV (↓)**	**ITH (↓)**	**BR_T (↑)**	
VCCS	82.59	590.77	20.26	5.32	
VCCS_KNN	90.55	320.69	14.82	18.47	
Octree	86.99	441.7	15.95	9.4	
SLIC	82.69	587.65	20.01	4.83	
LSC	81.13	640.35	20.42	4.84	
TBPS	84.93	511.43	18.38	6.64	
Ours	**94.04**	**202.23**	**13.31**	**36.53**	

**Table 2:** Performance comparison of object-level segmentation methods.

**Method**	**Standard Segmentation Metrics**							
	**BR_G (↑)**	**ASA (↑)**	**UE (↓)**	**VI (↓)**	**Prec (↑)**	**Rec (↑)**	**F1 (↑)**	**IoU (↑)**
EC	1.72	91.54	18.09	**0.43**	85.53	**99.99**	**92.2**	**85.52**
RG	**65.82**	47.04	**0**	1.32	93.42	20.63	33.79	20.33
Normal-based	60.43	76.86	7.68	0.83	85.37	69.57	76.67	62.16
LCCP (Graph)	3.36	93.04	23.04	5.07	84.73	7.07	13.05	6.98
Octree + RG	7.41	95.43	10.52	5.56	93.42	4.28	8.18	4.26
SLIC + RG	2.17	93.34	15.79	4.88	92.41	4.79	9.11	4.77
LSC + RG	2.68	93.19	14.71	5.01	93.59	4.52	8.61	4.5
Ours	27.66	**95.96**	7.45	4.34	**97.94**	23.33	37.69	23.22
**Method**	**Thermal-Specific Metrics**							
	**EV_temp (↑)**		**WTV (↓)**		**ITH (↓)**		**BR_T (↑)**	**BTC (↑)**
EC	1.77		3334.07		**6.15**		0.16	0.91
RG	8.91		3091.8		20.07		**75.28**	0.76
Normal-based	1.96		3327.47		13.21		54.89	1.07
LCCP (Graph)	62.98		1256.5		22.61		3.62	1.07
Octree + RG	79.4		699.04		18.55		5.46	0.93
SLIC + RG	73.35		904.39		27.73		2.78	0.93
LSC + RG	72.26		941.66		28.27		2.02	0.92
Ours	**89.32**		**362.49**		14.67		28.91	**1.33**

**Table 3:** Performance comparison of baseline algorithm with adapted thermal features.

**Method**	**Standard Segmentation Metrics**							
	**BR_G (↑)**	**ASA (↑)**	**UE (↓)**	**VI (↓)**	**Prec (↑)**	**Rec (↑)**	**F1 (↑)**	**IoU (↑)**
RG (color)	2.73	95.58	5.8	**0.3**	93.43	**99.84**	**96.53**	**93.29**
LSC (temp)	**94.64**	89.48	35.29	8.02	86.1	0.44	0.88	0.44
VCCS (color) + RG	1.97	93.46	15.38	4.79	92.56	5.16	9.78	5.14
VCCS (color) + LCCP	3.11	93.04	32.07	5.16	87.44	7.27	13.42	7.19
TBPS (color) + RG	21.14	95.87	**5.38**	4.62	95.92	5.56	10.52	5.55
TBPS (color) + LCCP	22.38	95.44	6.54	3.45	92.97	39.43	55.37	38.29
Ours	27.66	**95.96**	7.45	4.34	**97.94**	23.33	37.69	23.22
**Method**	**Thermal-Specific Metrics**							
	**EV_temp (↑)**		**WTV (↓)**		**ITH (↓)**		**BR_T (↑)**	**BTC (↑)**
RG (color)	13.1		2949.61		37.48		6.28	**3.64**
LSC (temp)	55.73		1502.68		32.05		**98.82**	3.07
VCCS (color) + RG	73.18		910.46		27.86		2.54	0.91
VCCS (color) + LCCP	58.47		1409.6		22.71		3.76	1.06
TBPS (color) + RG	85		509.14		17.14		19.53	1.25
TBPS (color) + LCCP	39.77		2044.35		15.13		18.95	1.26
Ours	**89.32**		**362.49**		**14.67**		28.91	1.33

## Data Availability

The data presented in this study are available on request from the corresponding author. The data are not publicly available due to privacy.
